# Integrated Data Analysis of Six Clinical Studies Points Toward Model-Informed Precision Dosing of Tamoxifen

**DOI:** 10.3389/fphar.2020.00283

**Published:** 2020-03-31

**Authors:** Lena Klopp-Schulze, Anna Mueller-Schoell, Patrick Neven, Stijn L. W. Koolen, Ron H. J. Mathijssen, Markus Joerger, Charlotte Kloft

**Affiliations:** ^1^Department of Clinical Pharmacy and Biochemistry, Institute of Pharmacy, Free University of Berlin, Berlin, Germany; ^2^Graduate Research Training Program PharMetrX, Berlin, Germany; ^3^Vesalius Research Center, University Hospitals Leuven, Katholieke Universiteit Leuven, Leuven, Belgium; ^4^Department of Medical Oncology, Erasmus MC Cancer Institute, Rotterdam, Netherlands; ^5^Department of Medical Oncology and Hematology, Cantonal Hospital, St., Gallen, Switzerland

**Keywords:** tamoxifen, modeling, simulation, pharmacokinetics, individualized dosing, model-informed precision dosing

## Abstract

**Introduction:**

At tamoxifen standard dosing, ∼20% of breast cancer patients do not reach proposed target endoxifen concentrations >5.97 ng/mL. Thus, better understanding the large interindividual variability in tamoxifen pharmacokinetics (PK) is crucial. By applying non-linear mixed-effects (NLME) modeling to a pooled ‘real-world’ clinical PK database, we aimed to (i) dissect several levels of variability and identify factors predictive for endoxifen exposure and (ii) assess different tamoxifen dosing strategies for their potential to increase the number of patients reaching target endoxifen concentrations.

**Methods:**

Tamoxifen and endoxifen concentrations with genetic and demographic data of 468 breast cancer patients from six reported studies were used to develop a NLME parent-metabolite PK model. Different levels of variability on model parameters or measurements were investigated and the impact of covariates thereupon explored. The model was subsequently applied in a simulation-based comparison of three dosing strategies with increasing degree of dose individualization for a large virtual breast cancer population. Interindividual variability of endoxifen concentrations and the fraction of patients at risk for not reaching target concentrations were assessed for each dosing strategy.

**Results and Conclusions:**

The integrated NLME model enabled to differentiate and quantify four levels of variability (interstudy, interindividual, interoccasion, and intraindividual). Strong influential factors, i.e., CYP2D6 activity score, drug–drug interactions with CYP3A and CYP2D6 inducers/inhibitors and age, were reliably identified, reducing interoccasion variability to <20% CV. Yet, unexplained interindividual variability in endoxifen formation remained large (47.2% CV). Hence, therapeutic drug monitoring seems promising for achieving endoxifen target concentrations. Three tamoxifen dosing strategies [standard dosing (20 mg QD), CYP2D6-guided dosing (20, 40, and 60 mg QD) and individual model-informed precision dosing (MIPD)] using three therapeutic drug monitoring samples (5–120 mg QD) were compared, leveraging the model. The proportion of patients at risk for not reaching target concentrations was 22.2% in standard dosing, 16.0% in CYP2D6-guided dosing and 7.19% in MIPD. While in CYP2D6-guided- and standard dosing interindividual variability in endoxifen concentrations was high (64.0% CV and 68.1% CV, respectively), it was considerably reduced in MIPD (24.0% CV). Hence, MIPD demonstrated to be the most promising strategy for achieving target endoxifen concentrations.

## Introduction

Even after 40 years since approval, tamoxifen remains one of the most important and frequently used oral anticancer drugs in the treatment of all stages of estrogen receptor-positive (ER+) breast cancer. Despite the therapeutic success in many breast cancer patients, unfortunately some patients do not respond or suffer from loss of response (Davies et al., 2013). High interindividual variability is observed in the PK of tamoxifen – and especially its active metabolite endoxifen – after standard doses of 20 mg tamoxifen once daily (QD). This variability has been partly attributed to genetic variations in the metabolizing enzyme CYP2D6 (Schroth et al., 2007; Mürdter et al., 2011; Ratain et al., 2013; Ter Heine et al., 2014). The variable endoxifen exposure is likely to contribute to the observed differences in clinical outcome and yet the ‘one-dose-fits-all’ treatment strategy is common practice. In the last decade, several clinical studies on tamoxifen treatment individualization have shown controversial results (Goetz et al., 2005; Barginear et al., 2011; Irvin et al., 2011; Regan et al., 2012; Martinez et al., 2014; Dezentjé et al., 2015; Welzen et al., 2015; Hertz et al., 2016; DeCensi et al., 2019; Khalaj et al., 2019) and caused intensive debates (Hoskins et al., 2009; Lash et al., 2009; Brauch et al., 2013; Binkhorst et al., 2015b).

This inevitably introduced uncertainty rather than solutions, by means of clear and useful guidance on how to make tamoxifen dosing decisions in clinical practice (Goetz et al., 2018). However, there seems to be considerable consensus that endoxifen, a secondary metabolite of tamoxifen, is an important driver of tamoxifen efficacy – being 100-fold more potent than its parent tamoxifen (Johnson et al., 2004; Kisanga et al., 2004; Lim et al., 2005; Mürdter et al., 2011; Gjerde et al., 2012). Two clinical studies have proposed a therapeutic threshold concentration of endoxifen in plasma which has been related to improved clinical outcome, i.e., a 26% reduced risk of breast cancer recurrence, longer distant relapse-free survival and reduced breast cancer-related death (Madlensky et al., 2011; Saladores et al., 2015). Applying this threshold, about 1 out of 5 patients will not reach therapeutic endoxifen concentrations under tamoxifen standard dosing and are thus at risk of treatment failure (Madlensky et al., 2011). Tamoxifen is still frequently used in pre- and postmenopausal ER+ breast cancer patients, and methods to achieve endoxifen plasma concentrations within the therapeutic target in all patients are of high interest.

Model-informed precision dosing (MIPD) aims to tailor doses to patients’ needs, and is therefore a promising tool to increase treatment success (Darwich et al., 2017). In this approach, patient characteristics are combined with information about drug pharmacokinetics (PK) and knowledge about exposure-response relationships in a comprehensive model-informed dose selection framework. While MIPD is increasingly applied in other therapeutic areas, i.e., anti-infective therapy (Darwich et al., 2017), it is virtually absent in oncology. Given the complex PK of tamoxifen, i.e., long time to steady-state (up to 1 month for tamoxifen, up to 3 months for endoxifen), extensive metabolism involving several polymorphic enzymes and a high potential for drug–drug interactions (DDIs), a model-informed approach seems particularly suitable to capture influential factors and quantify their impact on the PK of tamoxifen and endoxifen.

Prior to conducting a ‘real’ clinical study, it is useful to circumvent the many limitations associated with a clinical trial and first explore the impact of the proposed intervention using a less time- and resource-consuming *in silico* approach. The *in silico* approach further allows to study scenarios which would be challenging and/or time consuming to observe in ‘real-life,’ due to the rareness of subpopulations (i.e., CYP2D6 poor metabolizer) or ethical concerns (investigating doses outside the approved dose range).

Based on this *status quo*, the aim of this work was (i) to enhance the quantitative understanding of the highly variable pharmacokinetics and pharmacogenetics of tamoxifen and its major active metabolite endoxifen and (ii) to investigate intrinsic and extrinsic factors causing subtarget endoxifen plasma concentrations in order (iii) to propose a possible clinical trial design [i.e., how many therapeutic drug monitoring (TDM) samples should be taken and when] to evaluate the clinical benefit of model-informed precision dosing of tamoxifen. To that end, first a large and modular ‘clinical PK database’ for modeling & simulation (M&S) analyses of tamoxifen and its metabolites was created by pooling six clinical studies, enabling subsequent integrated data analyses utilizing pharmacometric approaches. Secondly, a joint non-linear mixed-effects pharmacokinetic (NLME-PK) model of tamoxifen and endoxifen was developed and evaluated by integrating information on clinical pharmacokinetics, pharmacogenetics and CYP-mediated drug–drug interactions from the established PK database. Thirdly, within the NLME-PK model, multiple levels of random variability in the most relevant PK processes were dissected, quantified and characterized to determine the magnitude of ‘real-world’ variability and to detect sources of PK variability (patient- and treatment-specific characteristics), thereby identifying patients at risk of subtarget endoxifen concentrations. Finally, the NLME-PK model was applied to investigate and compare three different dosing strategies, namely standard dosing, CYP2D6-adapted dosing and MIPD. Clinical trial simulations were used to demonstrate advantages and disadvantages of the respective strategies and thus guide toward a more appropriate, individualized treatment strategy.

## Materials and Methods

### Clinical PK Database of Tamoxifen and Endoxifen

A single ‘clinical PK database’ was generated by pooling multiple clinical datasets from six reported clinical investigator-initiated tamoxifen trials [hereinafter referred to as studies 1–6 (de Graan et al., 2011; Binkhorst et al., 2012, 2015a, 2016; Poppe et al., 2016; Neven et al., 2018)], all carried out in line with the recommendations of the World Medical Association’s Declaration of Helsinki and their study protocols approved by the respective ethics committee. All patients gave written informed consent prior to participation. Each study contributed relevant clinical pharmacokinetic, pharmacogenetic and demographic information representing clinical routine, “real-world” data. ‘Real-world’ data refer to data from non-randomized controlled trials, e.g., clinical investigator-initiated trials and observational cohort studies, which reflect a real-world scenario (Franklin et al., 2019). The database assembled an extensive collection of plasma concentration data of tamoxifen and its major metabolite endoxifen (*n*_*PK observations*_ = 3554) and a variety of patient information from 468 breast cancer patients (Table 1). Due to the original objectives of the single studies, respective study designs, study population sizes and blood sampling frequencies differed (Table 1). Inclusion and exclusion criteria, analytical methods and treatment settings for each study are provided in the Supplementary Material (see section “Extended Information on the Six Clinical Tamoxifen Studies Featured in the Clinical PK Database”).

**TABLE 1:** Study characteristics of the clinical PK database of six pooled tamoxifen studies.

	**Study 1 (Neven et al., 2018)**	**Study 2 (Poppe et al., 2016)**	**Study 3 (de Graan et al., 2011)**	**Study 4 (Binkhorst et al., 2012)**	**Study 5 (Binkhorst et al., 2016)**	**Study 6 (Binkhorst et al., 2015a)**	**Total**
*N*_P__atients_	247	128	40	7	15	31	468
*N*_Samples_	405	128	345	180	238	639	1935
*N*_PK observations_	810	256	690	364	478	956	3554
*N*_S__amples/pt__(s)_	≤4	1	≤9	≤ 18	≤18	≤ 27	–
*N*_S__ampling periods_	≤ 4	1	1	≤ 3	≤3	≤3	–
20 mg QD, %pts	100	100	70	100	73	97	96
40 mg QD, %pts	0	0	30	0	27	3	4
TSFD [months], median (range)	3.4 (0.14–11)	5.6 (2.2–10)	9.5 (0.32–70)	10.4 (6.6–47)	2.3 (1.1–57)	7.3 (1.0–35)	–
Setting, %pts adjuvant/neo-adjuvant/metastatic	25 75	100	n.r. (5) 65 30	100	n.r. (33) 67	n.r. (3) 97	n.r. (2) 43 13 42
Age [years], median (range)	72 (48–95)	61 (41–80)	52 (25–70)	51 (29–60)	51 (38–65)	51 (27–68)	64 (25–95)
CYP2D6, %pts							
gUM	0	5	0	0	0	0	1
gNM	82	69	83	14	47	87	78
gIM	10	12	10	0	7	0	8
gPM	5	7	3	14	0	10	6
n.r.	3	8	4	72	47	3	7
SSRI, %pts	2.0	3.9	2.5	0	80	0	4.9
RIF, %pts	0	0	0	70	0	0	0.01

**gUM, gNM, gIM, gPM: CYP2D6 genotype-predicted ultrarapid, normal, intermediate and poor metabolizer, n.r.: not reported; PK observations: plasma concentrations of tamoxifen and endoxifen; pt(s): patient(s); QD: dosing once daily; SSRI: concomitant use of fluoxetine or paroxetine (selective serotonin reuptake inhibitors); RIF: concomitant use of rifampicin; TSFD: time since first dose*.*

#### Pharmacogenetics: CYP2D6 Genotype-to-Phenotype Predictions

**FIGURE 1:**
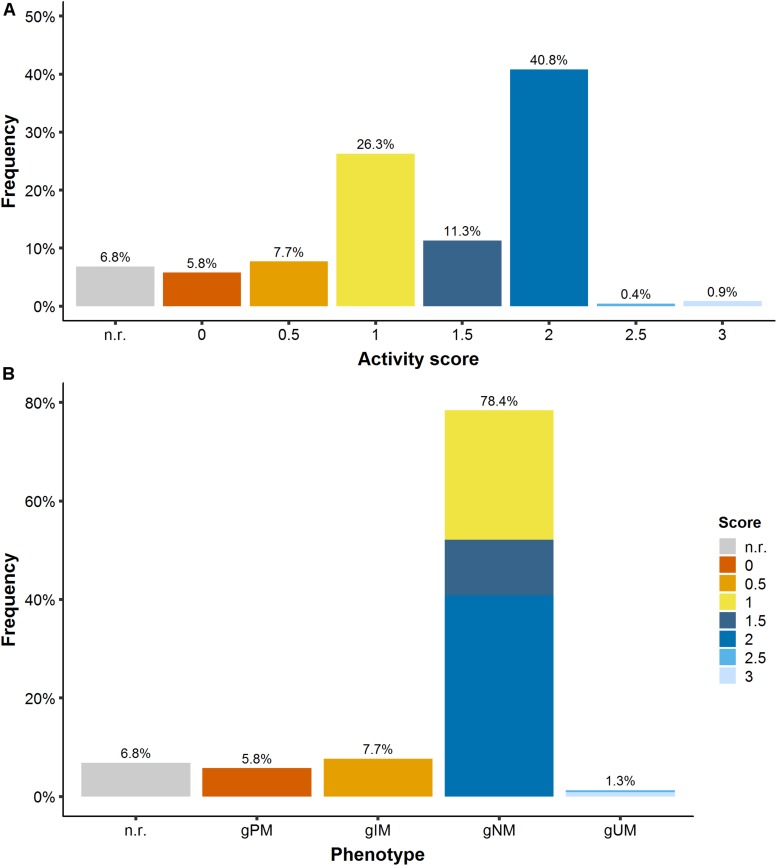
**(A)** CYP2D6 activity score frequency and **(B)** translation into CYP2D6 phenotype. *gPM/gIM/gNM/gUM*: poor/intermediate/normal and ultrarapid metabolizer predicted from genotype; *n.r.*, not reported.

To derive a patient’s CYP2D6 phenotype, the available genotype information, i.e., detected genetic polymorphisms (single nucleotide polymorphisms, SNPs, and copy number variants, CNVs), was translated via three steps: (1) From the reported nucleotide variation (wild-type ‘wt’ or altered nucleotide sequence ‘mut’) to a biallelic genotype, e.g., wt/mut: ^∗^1/^∗^41; (2) from the genotype to the activity score (AS), e.g., ^∗^1/^∗^41 → 1.5; (3) From the AS to the ‘traditional’ CYP2D6 phenotype category, e.g., 1.5 → normal metabolizer, gNM. The activity score assignment was performed based on the Clinical Pharmacogenetics Implementation Consortium (CPIC) guideline (Gaedigk et al., 2017; Goetz et al., 2018). The CPIC is an international consortium of experts developing evidence-based gene/drug clinical practice guidelines (CPIC, 2019) and therapeutic recommendations. Thus, each patient in the PK database was assigned a CYP2D6 activity score (seven AS categories from 0 to 3 in intervals of 0.5) and a respective CYP2D6 phenotype [four categories: poor (gPM, AS: 0), intermediate (gIM, AS: 0.5), normal (gNM, AS:1-2), and ultrarapid (gUM, AS ≥ 2) metabolizer; see Figure 1]. The prefix ‘g’ indicates the phenotype prediction from genotype.

### Non-linear Mixed-Effects PK Model Approach to Dissect, Quantify and Characterize Multiple Levels of Variability

For the purpose of better understanding the – unexplainable and explainable – variability in tamoxifen and endoxifen exposure, and to address questions concerning potential tamoxifen dose optimization strategies, a joint parent-metabolite model focusing on key covariate-parameter relationships was developed.

#### Structural Model Development

The structural submodel aimed to describe the general trend in the observed data of both tamoxifen and its most active metabolite endoxifen using a system of ordinary differential equations. One-compartment as well as two-compartment models for tamoxifen with first- or zero-order absorption with or without lag-time and linear or non-linear clearance as well as endoxifen formation were evaluated. The structural model describing the observed tamoxifen and endoxifen concentrations best was carried forward for the development of the statistical submodel. PK parameters for tamoxifen and endoxifen were simultaneously estimated.

#### Statistical Submodel Development

The statistical model aimed to incorporate four levels of variability around the structural model. Thus, and due to the nature of the database, a nested hierarchy of random-effects components was set up comprising interstudy (ISV), interindividual (IIV), interoccasion (IOV), and residual unexplained variability (RUV) (see Supplementary Figure S1). While ISV and IIV characterize random deviations between different studies and patients, respectively, IOV describes the random variability within an individual patient across different occasions. The RUV captures the remaining variability across all observations.

IIV was evaluated on all structural PK parameters and implemented using exponential IIV models (Equation 1). Resulting individual PK parameters were assumed to arise from a log-normal distribution in which the η_*ik*_ values are normally distributed with mean zero and variance estimate $\omega_{\text{k}}^{2}$.

(1) $$\theta_{\text{ik}}=\theta_{\text{k}}\cdot e^{\eta_{\text{ik}}}\;\eta_{\text{ik}}∼𝒩\,\left(0,\omega_{\text{k}}^{2}\right)$$

with typical PK parameter θ_*k*_ and patient individual PK parameter θ_*ik*_for patient *i* = 1,…,*N* and PK parameter *k* = 1,…,*N*.

While 27% of the patients had samples collected on one occasion (study day, i.e., one dosing interval = 1 day) only, 73% of patients in the PK database had samples collected on three to four occasions (i.e., 3–4 days) with rich sampling during one occasion (dosing interval) in studies 3–6. Hence, IOV was considered on PK parameters for these four different occasions (Equation 2).

(2) $$\theta_{\text{ikq}}=\theta_{\text{k}}\cdot e^{\eta_{\text{ik}}+\kappa_{\text{ikq}}}\,\kappa_{\text{ikq}}∼𝒩\,\left(0,\pi_{\text{k}}^{2}\right)\,\mathrm{for}\,\mathrm{the}\,\mathrm{qth}\,\mathrm{occasion}.$$

with typical PK parameter θ_*k*_ and patient individual PK parameter θ_*ikq*_for patient *i* = 1,…,*N*, PK parameter *k* = 1,…,*N* and occasion *q* = 1,…,*N*.

For the RUV, a log-transformed both sides approach was applied (Equation 3).

$$\ln\left(Y_{\text{ij}}\right)=\ln(f\left(x_{\text{ij}},\theta_{\text{i}}\right)\cdot e^{\varepsilon_{\text{ij}}}\;\varepsilon_{\text{ij}}∼𝒩\left(0,\sigma_{\text{ij}}^{2}\right)$$

(3) $$\ln\left(Y_{\text{ij}}\right)=\ln(f\left(x_{\text{ij}},\theta_{\text{i}}\right)+\varepsilon_{\text{ij}}\;\text{with}\varepsilon_{\text{ij}}=Y_{\text{ij}}^{\text{obs}}-Y_{\text{ij}}^{\text{pred}}.$$

Since tamoxifen and endoxifen PK were analyzed simultaneously, separate but correlated RUV terms were estimated. As only in one patient tamoxifen was quantified but endoxifen was not, sufficient parent metabolite concentration pairs to allow precise estimation of both RUV terms and their correlation were available.

A study effect was introduced as additional variability term on top of the random individual effect (IIV) (study hierarchically above individual level, see Supplementary Figure S1 and Equation 4) (Laporte-Simitsidis et al., 2000),

$$\theta_{\text{ks}}=\tilde{\theta_{\text{k}}}\cdot e^{\gamma_{\text{ks}}}\;\gamma_{\text{ks}}∼𝒩\,\left(0,\varphi_{\text{k}}^{2}\right)$$

(4) $$\theta_{\text{ksi}}=\theta_{\text{ks}}\cdot e^{\eta_{\text{ik}}}\;\eta_{\text{ik}}∼𝒩\,\left(0,\omega_{\text{k}}^{2}\right)$$

with typical population estimate $\tilde{\theta_{\text{k}}}$ of PK parameter *k*, deviation γ_ks_ between $\tilde{\theta_{\text{k}}}$ and the respective study parameter θ_*ks*_ and η_*ik*_the deviation between θ_*ks*_ and the individual PK parameter θ_ksi_.

#### Covariate Submodel Development

The covariate model was developed using a full covariate model approach (Tunblad et al., 2008; Ravva et al., 2009; Gastonguay, 2011): first, covariates were pre-selected based on specific criteria and introduced simultaneously into a full covariate model (for details see Supplementary Material 1, see section “Covariate Submodel Development”). Secondly, a covariate model refinement step was performed, selecting the most appropriate covariate functions for the preselected covariates, with respect to numerical and statistical evaluation criteria. Finally, the refined full covariate model was evaluated based on physiological plausibility of the estimated effect, statistical significance and clinical relevance criteria using advanced model evaluation techniques (Supplementary Material 1, see section “Advanced Model Evaluation Diagnostics”). To explore and quantify the reduction of unexplained variability upon introduction of covariate effects, the statistical model with the four levels of variability was exploited.

#### Determination of Patients at Risk of Subtarget Endoxifen Concentrations

To determine the probability of endoxifen target attainment (PTA) according to (Madlensky et al., 2011) for different patient subgroups, tamoxifen and endoxifen concentration-time profiles in a large tamoxifen patient population (*n*_*replicates*_ = 1,000 of the original database) were simulated using the final developed model without ISV. After stratification into the respective subgroups based on CYP2D6 genotype-predicted phenotype and comedication, the percentage of patients at risk for subtarget endoxifen concentrations was determined per subgroup (Equation 5).

(5) $$Riskpergroup,\%=\frac{N\,u\,m\,b\,e\,r\,o\,f\,p\,a\,t\,i\,e\,n\,t\,s\,p\,e\,r\,s\,u\,b\,g\,r\,o\,u\,p<C_{\mathrm{TH},\mathrm{ENDX}}}{T\,o\,t\,a\,l\,n\,u\,m\,b\,e\,r\,o\,f\,p\,a\,t\,i\,e\,n\,t\,s\,p\,e\,r\,s\,u\,b\,g\,r\,o\,u\,p}\cdot 100$$

with *C*_*TH*,*ENDX*_ = endoxifen minimum concentrations at steady-state below the endoxifen target concentration 5.97 ng/mL (Madlensky et al., 2011).

The simulated risk values were compared to the corresponding observed risk values in the tamoxifen PK database.

### PK Model Applications: *In silico* Clinical Studies of Different Dosing Strategies

#### Standard vs. Two More Individualized Tamoxifen Dosing Strategies

**FIGURE 2:**
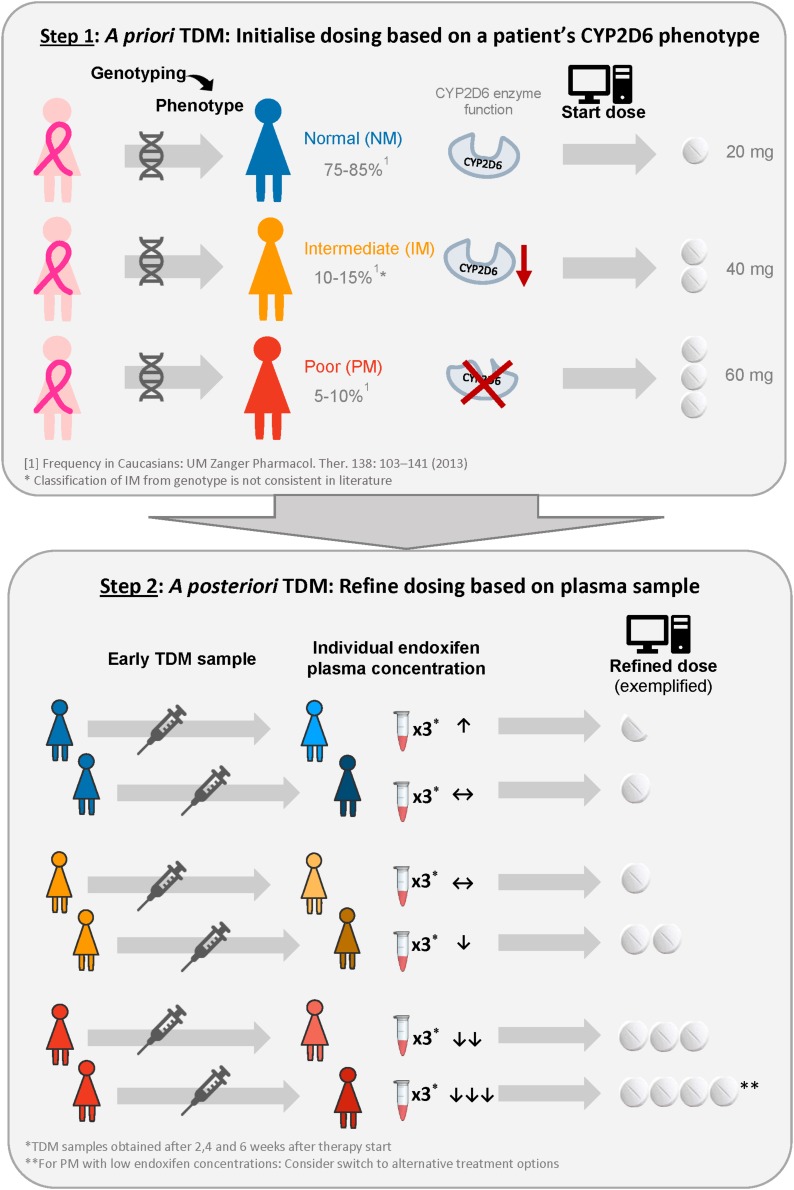
Illustration of the proposed model-informed precision dosing strategy to provide each patient with the lowest required tamoxifen dose to reach the target concentration. ↑, ↔, ↓ indicate that minimum endoxifen plasma concentrations at steady-state are well above, above and below the therapeutic target concentration (Madlensky et al., 2011).

The developed model was applied in an *in silico* simulation study to compare three dosing strategies which could subsequently be assessed in a clinical trial: (1) Standard dosing (20 mg tamoxifen QD), (2) CYP2D6-guided dosing, and (3) model-informed precision dosing (Figure 2). Prior knowledge was used in CYP2D6-guided dosing, in which the daily tamoxifen dose was selected based on a patient’s CYP2D6 functional activity (*a priori* model-informed dosing, i.e., in absence of measured PK concentrations). Respective doses were selected such that each CYP2D6 genotype-predicted phenotype subgroup would achieve therapeutic target concentrations comparable to gNM under tamoxifen standard dosing (20 mg QD). In MIPD, after an initial CYP2D6-guided dosing period of 4 weeks, the Bayesian approach was applied to ‘forecast’ an individual maintenance dose: concretely, the new individual PK information derived from three virtual TDM samples after weeks 2, 3, and 4 was combined with patient characteristics CYP2D6 phenotype and age to estimate patients’ individual PK parameters using the developed model to further tailor the dose to a patient’s needs (*a posteriori* model-informed dosing). The chosen frequency and time points of virtual TDM sampling were the result of previous simulation-based investigations aiming to find a schedule which would simultaneously minimize (i) the frequency of patients for whom too low doses are selected and (ii) the time until target concentrations are reached. Applying Bayes’ formula, individual most likely PK model parameter estimates [maximum *a posteriori* (MAP) PK parameter estimates] for every patient, given the patient’s endoxifen observations, CYP2D6 phenotype and the PK parameter distribution of the developed model were derived by minimizing Equation 6. For illustration purposes, the PK parameters and endoxifen observations are described using the normal distribution assumption, while in the presented simulation study log-transformed parameter and concentration values were used.

(6) $$O\,F\,V_{\text{MAP}}=\sum_{j=1}^{m}\frac{{\left(C_{\mathrm{obs},\mathrm{ij}}-{\hat{C}}_{\text{ij}}\right)}^{2}}{\sigma^{2}}+\sum_{k=1}^{n}\frac{{\left(\theta_{\text{ki}}-\theta_{\text{k}}\right)}^{2}}{\omega_{\text{k}}^{2}}$$

In Equation 6, the left term on the right hand side describes the sum of the squared deviations of the individual model-predicted concentrations ${\hat{C}}_{\text{ij}}$ from the observed concentrations *C*_*obs,ij*_ of individual *i*at time point *j*, which are weighted by the residual variance σ^2^ of the underlying RUV model. The right term describes the sum of the squared deviations of the individual PK parameter estimates θ_*ki*_ from the typical population parameter values θ_*k*_ of individual *i*at time point *k*, which are weighted by the interindividual variance $\omega_{\text{k}}^{2}$ of parameter *k* of the underlying IIV model. Thus, the left term representing the likelihood of observing the PK sample is balanced by the right term representing the prior knowledge on PK parameter distributions. Consequently, the more information (i.e., TDM samples) is available from a patient, the more influential the left term on the right hand side of the equation, so that individual observations *C*_*obs,ij*_ increasingly minimize the influence of the typical population parameter values. Individual plasma concentrations were predicted for every patient using individual MAP PK parameter estimates for the dose range between 5 and 120 mg QD and the lowest required dose to reach endoxifen target threshold concentrations at steady-state was selected for each patient. This range of maintenance tamoxifen dose levels (5–120 mg QD) was based on the lowest available dose in tablet form and the highest dose applied in a clinical trial, respectively (Dezentjé et al., 2015).

Prior to the simulations, the full covariate model was modified as such that IOV terms were removed and model parameters re-estimated, resulting in slightly inflated IIV and RUV components. For all simulations, a large virtual patient population (*n* = 10,000), representing the distribution of patient characteristics age and CYP2D6 AS in the clinical PK database, was used. The age distribution in the virtual patient population was generated using a truncated normal distribution based on the observed age values in the clinical PK database with minimum (25 years) and maximum (95 years) observed ages as cutoff points, respectively. CYP2D6 genotype frequencies were extrapolated from the clinical PK database. No interacting comedication such as SSRIs or rifampicin was assumed to be administered. The high number of virtual patients facilitated to observe sufficiently high numbers of patients with rare CYP2D6 genotypes (e.g., poor and ultrarapid metabolizers) as well as the ‘real-world’ variability in a typical breast cancer population.

For all dosing strategies, the numbers of patients at risk for subtarget endoxifen concentrations after 6 months tamoxifen treatment were compared overall and for CYP2D6 genotype-predicted phenotypes separately. Furthermore, the interindividual variability in endoxifen concentrations within the patient population was assessed for and compared between all dosing strategies.

## Results

### Clinical PK Database of Tamoxifen and Endoxifen

The PK database contained *C*_*ss,minTAM*_ and *C*_*ss,minENDX*_ from 468 adult, female breast cancer patients (see Table 1). Only eight endoxifen concentration values (of a single patient, 0.20% of all observations) were below the lower limit of quantification and therefore excluded from the analysis. Tamoxifen concentration values of this patient were still included in the analysis to inform tamoxifen PK parameters. Most patients (96%) had been treated with 20 mg tamoxifen QD (standard treatment). Five patients had temporarily co-administered rifampicin (a strong CYP3A inducer) and 23 patients had switched from paroxetine or fluoxetine (SSRIs) comedication (potent CYP2D6 inhibitors) to es/citalopram (antidepressant with no relevant drug–drug interaction with tamoxifen (Binkhorst et al., 2016).

**FIGURE 3:**
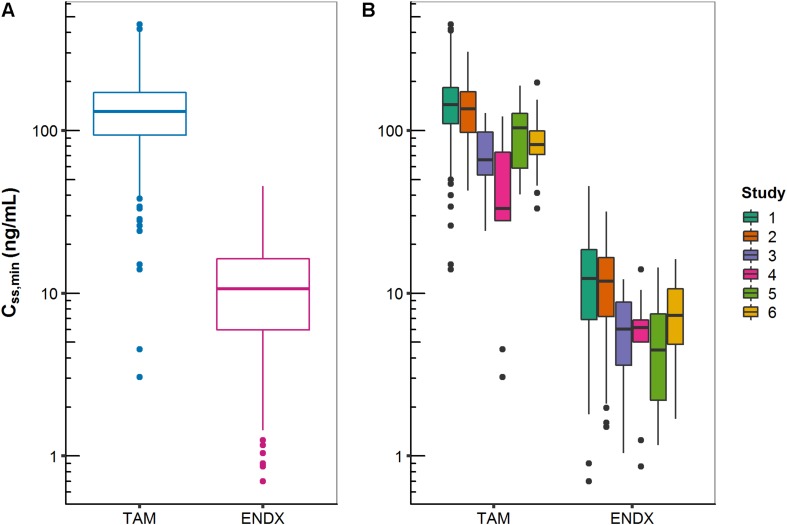
Minimum concentrations of tamoxifen (TAM) and endoxifen (ENDX) at steady-state (*C*_*SS,min*_) in patients treated with 20 mg tamoxifen once daily: **(A)** all studies combined and **(B)** stratified by study.

Across all studies, median *C*_*ss,minTAM*_ and *C*_*ss,minENDX*_ (of the 20 mg dose group) were 131 and 10.7 ng/mL and ranged widely from 3.05–448 to 0.70–45.5 ng/mL (i.e., %CVgeom of 56.3 and 84.4), respectively (Figure 3A). Considerable differences in median *C*_*SS,min*_ were revealed after stratification into studies (Figure 3B); which were in part expected (i.e., studies 4 and 5 which investigated drug–drug interactions, CYP3A induction and CYP2D6 inhibition, on PK, respectively).

**FIGURE 4:**
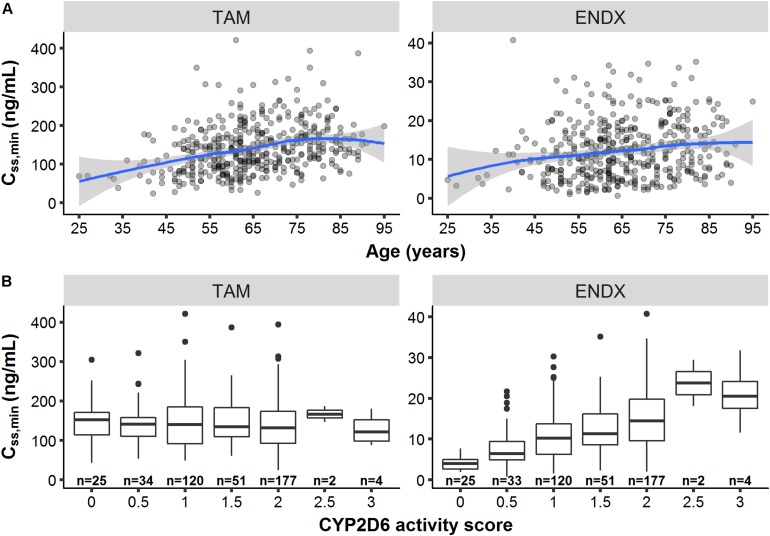
Minimum concentrations of tamoxifen (TAM) and endoxifen (ENDX) at steady-state (*C*_*SS,min*_) at the first visit (*n* = 440 patients) **(A)** across age and **(B)** CYP2D6 activity scores. Patients with rifampicin or paroxetine/fluoxetine comedication were excluded (*n* = 14). Patients with missing age (*n* = 6) and missing CYP2D6 genotype data (*n* = 27) were additionally excluded for **(A,B)**, respectively. Solid line: smoothing spline; Area around spline: 95th confidence interval.

Besides the influence of the DDIs (with rifampicin and SSRIs) on *C*_*ss,minTAM*_ and *C*_*ss,minENDX*_ (Figure 3B), exploratory graphical analyses of the PK database displayed that *C*_*ss,minTAM*_ (and *C*_*ss,minENDX*_) increased with age (Figure 4A), however, *C*_*SS,min*_ were widely scattered. In addition, *C*_*ss,minENDX*_ increased with increasing CYP2D6 activity, displayed by the ordered categorical covariate CYP2D6 activity score (Figure 4B).

The PK database was representative of a Caucasian CYP2D6 population (Gaedigk et al., 2008) containing 5.8% and 1.3% of the rare gPM (AS: 0) and gUM (AS: 2.5–3), respectively (Figure 1).

Patients without CYP2D6 information (*n* = 32, 6.84%) were classified as normal metabolizers (i.e., CYP2D6 AS: 2), representing the most frequent group in a Caucasian population and thus, the reference category. For patients with missing age information (*n* = 10, 2.14%), the population median was imputed. For missing comedication data (*n* = 376, 80.3% for rifampicin; *n* = 1, 0.214% for SSRIs), no comedication was assumed.

### Joint PK Model and Characterization of Influential Factors on Tamoxifen and Endoxifen PK

**FIGURE 5:**
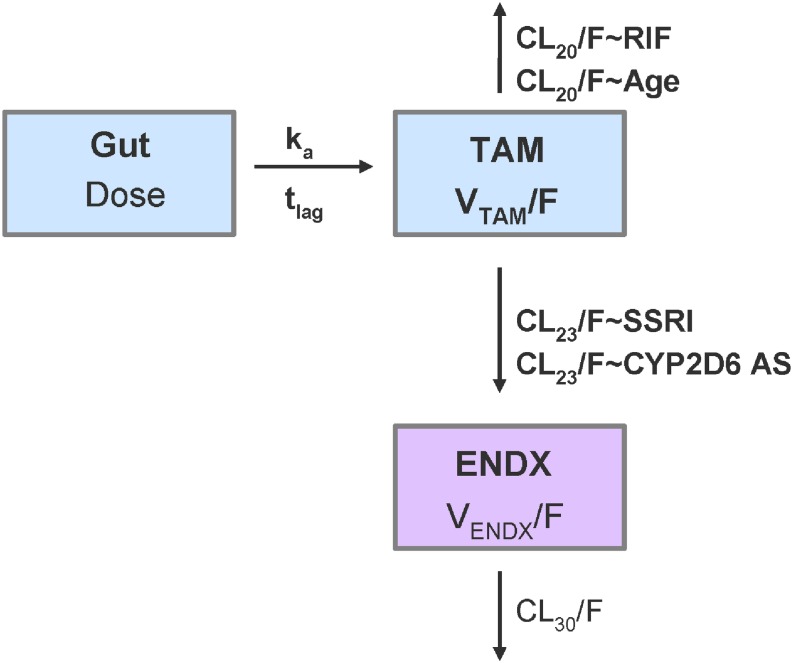
Schematic representation of the joint tamoxifen and endoxifen PK model including covariate relationships. CL_20_/F: relative clearance of tamoxifen; CL_23_/F: relative formation of endoxifen; CL_30_/F: relative clearance of endoxifen; CYP2D6 AS: CYP2D6 activity scores as ordered categorical covariate from 0 to 3 in increments of 0.5; ENDX: endoxifen compartment with *V_*ENDX*_/F;* k_*a*_: absorption rate constant; RIF: rifampicin comedication as categorical covariate (proportional change of respective structural PK parameter); SSRI: paroxetine/-fluoxetine comedication as categorical covariate (proportional change of respective structural PK parameter); Gut: tamoxifen dose in gut compartment; TAM_*C*_: central tamoxifen compartment with *V_*TAM*_/F*; t_*lag*_: lag time; V_*X*_/F: relative volume of distribution of *x*, being either tamoxifen or endoxifen; bold: estimated parameters [other parameters fixed to values from literature (Ahmad et al., 2010b)].

A one-compartment model with first-order absorption with lag time and first-order elimination for tamoxifen PK, linked to a one-compartment model with first-order formation (from tamoxifen compartment) and first-order elimination for endoxifen, was selected as appropriate and stable PK model with respect to its context of use (Figure 5). Since no intravenous data was available and hence absolute bioavailability (F) remained unknown, the PK model was parameterized in terms of relative clearances (CL20/F; CL23/F; and CL30/F) and relative central volume of distribution (V_*TAM*_/F; V_*ENDX*_/F). Furthermore, endoxifen PK parameter values (CL30/F = 5.1 L/h and V_*END*__*X*_/F = 400 L) were informed and used from a clinical study administering endoxifen only (Ahmad et al., 2010b).

**FIGURE 6:**
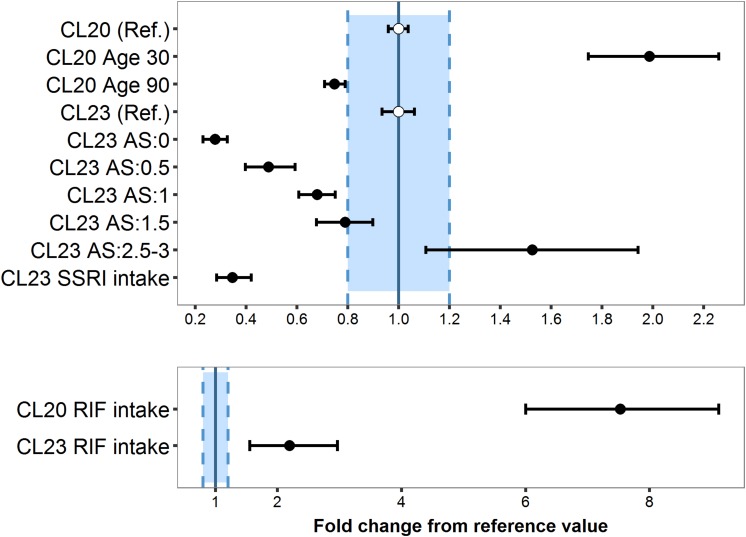
Magnitude and precision of covariate effects age, CYP2D6 activity score and selective serotonin -reuptake inhibitor (SSRI: paroxetine/fluoxetine; CYP2D6 inhibitors) **(A)** or rifampicin (RIF; CYP3A inducer) **(B)** on tamoxifen clearance (*CL_20_/F*) and endoxifen formation (*CL_23_/F)* relative to the reference patient (Ref.), i.e., CYP2D6 normal metabolizer (AS: 2), 65 years old, no coadministered RIF or SSRI.

Parameter estimates and their corresponding relative standard errors (RSEs) and confidence intervals (CIs), as measures of uncertainty, are summarized in Table 2, left. The typical population PK parameter estimates (95% CI) given the reference patient (CYP2D6 activity score 2/fast-normal metabolizer gNM-F, 65 years old, no comedication with rifampicin as CYP3A inducer or paroxetine/fluoxetine as selective serotonin-reuptake inhibitors and CYP2D6 inhibitors) were 5.77 L/h for CL20/F, 0.493 L/h for CL23/F and 1120 L for V_*TAM*_/F. All structural parameters were estimated with high precision (RSEs ≤ 16.5%). Individual information on CL20/F and CL23/F was sufficient to estimate IIV components and consequently, identify sources of variability. As both processes are mediated via different CYP enzymes (CL20/F mostly via CYP3A4, CL23/F mostly via CYP2D6), a correlation between both IIV components seemed unlikely. However, as both are competing processes, a correlation between the IIV parameters for CL20/F and CL23/F could be possible and was thus evaluated. Including the correlation term decreased the objective function value by 3.9 points. However, the correlation was low and resulted in increased relative standard errors in the structural parameters. Thus, it was decided to not include a correlation between IIV parameters for CL20/F and CL23/F. IIV components for V_*TAM*_/F, V_*ENDX*_/F, lag time and the absorption rate constant were not supported by the data. The effects of the preselected covariates age, CYP2D6 activity score and comedication on CL20/F and CL23/F, were strong (clinically relevant, s. Figures 6A,B) and precisely (RSE: 3–42%) identified:

**TABLE 2:** Parameter estimates of the joint parent-metabolite PK model of tamoxifen and endoxifen using the clinical PK database.

	**Parameter [unit]**	**Estimate**	**RSE,%**	**95% CI**	
**Fixed effects**	ka [1/h]	1.78	14.2	1.40	2.36
	t_lag_ [h]	0.389	5.80	0.344	0.431
	V_TAM_/F [L]	1120	16.4	861	1599
	CL30/F [L/h]	5.10 fixed			
	V_ENDX_/F [L]	400 fixed			
	CL20/F [L/h] (Ref.)	5.77	1.98	5.54	6.00
	V_TAM_/F_Rifampicin*	0.581	34.4	0.167	0.948
	CL20/F_Rifampicin*	6.51	11.8	5.00	8.11
	CL20/F_Age**	−0.886	9.73	–1.05	–0.72
	CL23/F [L/h] (Ref.)	0.493	3.17	0.461	0.524
	CL23/F_AS: 0*	−0.722	3.40	–0.769	–0.674
	CL23/F_AS: 0.5*	−0.510	10.2	–0.603	–0.406
	CL23/F_AS: 1*	−0.323	11.6	–0.392	–0.249
	CL23/F_AS: 1.5*	−0.211	26.6	–0.323	–0.099
	CL23/F_AS: 2.5-3*	0.533	41.4	0.108	0.944
	CL23_SSRI*	−0.654	5.32	–0.715	–0.578
	CL23_Rifampicin*	1.18	30.2	0.548	1.96
**Random effects**	IIV CL20/F	0.148 (39.9% CV)	8.98	0.122	0.172
	IIV CL23/F	0.201 (47.2% CV)	9.20	0.168	0.238
	IOV CL20/F	0.0222 (15.0% CV)	28.5	0.0133	0.0371
	IOV CL23/F	0.0289 (17.1% CV)	18.3	0.0209	0.0414
	RUV tamoxifen	0.0260 (16.2% CV)	5.76	0.0236	0.0295
	COVAR_RUVtam–RUVendx_	0.0169	7.28	0.0148	0.0195
	RUV endoxifen	0.0267 (16.5% CV)	6.19	0.0227	0.0291

*Fractional change covariate models, see Supplementary Material Equations 1 and 8; **Power covariate model, see Supplementary Material Equation 4; AS: CYP2D6 activity score from 0 to 3 with increments of 0.5; CI: 95% confidence interval for the estimates derived from sampling importance resampling method; COVAR: covariance; CL20/F: relative clearance of tamoxifen; CL23/F: relative formation of endoxifen; CL30/F: relative clearance of endoxifen; IIV: interindividual variability; IOV: Inter-occasion variability; ka: absorption rate constant; CV: %coefficient of variation of estimate; Fixed: parameter fixed based on data from a study administering endoxifen only (Ahmad et al., 2010b); Rifampicin: CYP3A inducer comedication; RSE: %relative standard error = (SE/Estimate)⋅100; RUV: residual unexplained variability; SSRI: fluoxetine or paroxetine comedication (CYP2D6 inhibitors); t_lag_: absorption lag time; V_TAM_/F, V_ENDX_/F: relative central volume of distribution of tamoxifen and endoxifen, respectively.

#### Age

The average population estimate for the power model effect of age on tamoxifen clearance CL20/F was −0.886 (95% CI: −1.06, −0.721) (Table 2), indicating CL20/F decreased with age: Compared to the 65 years old reference patient, the relative eliminating efficacy was twofold higher in patients of 30 years of age and 25% lower in patients of 90 years (Figure 6A).

#### CYP2D6 Activity Score

The lower the CYP2D6 activity score (AS), the lower the endoxifen formation CL23/F: Poor (AS: 0) and intermediate metabolizers (AS: 0.5) showed a 72.2% (95% CI: 67.4–76.9%) and 51.0% (95% CI: 40.6–60.3%) reduced formation efficiency, respectively, while ultrarapid metabolizer (AS ≥ 2.5) displayed a 53.3% (95% CI: 10.8–91.4%) increased CL23/F (Table 2). AS 1 and 1.5 displayed a fractional decrease from CL23/F reference value of 32.3% (95% CI: 24.9–39.2%) and 21.1% (95% CI: 9.94–32.3%), respectively.

Fractions of tamoxifen metabolized to endoxifen (FM), calculated as

(7) $$FM,\%=\frac{C\,L\,23/F}{\left(C\,L\,23/F+C\,L\,20/F\right)}\cdot 100$$

were 7.87% in patients with reference category AS 2 (gNM-F). In intermediate (AS: 0.5) and poor (AS: 0) metabolizers, the FM was reduced by 48.9 and 70.5%, respectively, while in ultrarapid metabolizers the FM was higher by 47.4%.

#### Comedication: CYP-Mediated Drug Interaction

Although the number of patients who had rifampicin or SSRIs coadministered was small (0.01 and 4.9% of the total population, respectively), the covariate effects were strong (clinically relevant, s. Figures 6A,B) and precisely (RSE: 13–42%) estimated using fractional change models: Rifampicin intake caused a substantial increase of 6.51-fold in CL20/F (95% CI: 5.00–8.11) and of 1.18-fold in CL23/F (95% CI: 0.548–1.97) (Figure 6B). Due to the high CL20/F during rifampicin coadministration, the FM (Equation 2) was substantially reduced to 2.42% in a typical NM (AS = 2) compared to a typical NM patient not taking rifampicin. SSRIs coadministration in a typical NM led to a 65.4% decrease in CL23/F (95%CI: 57.8–71.5%) (Table 2) translating into a low FM (2.89%).

### Joint PK Model Evaluation

**FIGURE 7:**
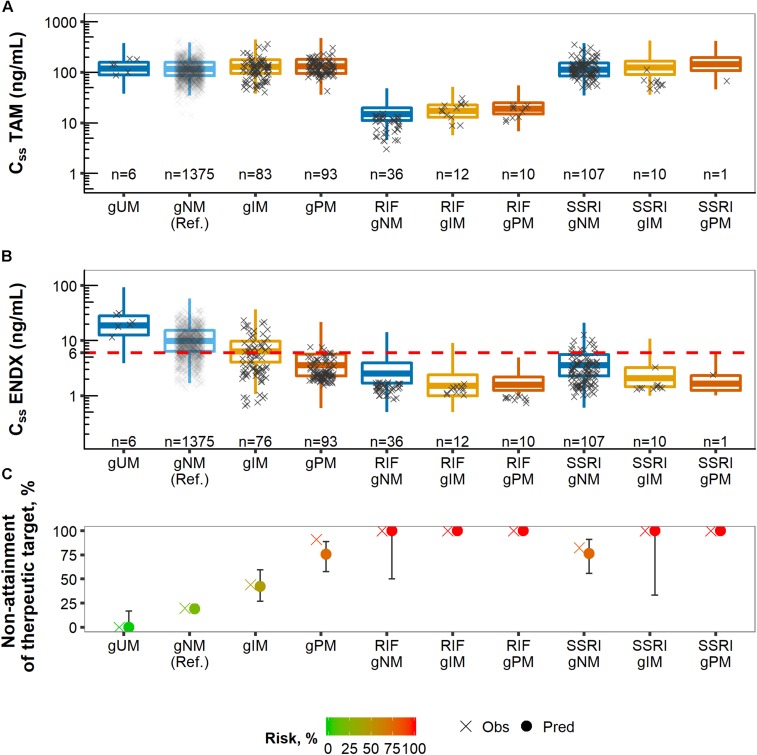
Predicted (boxes) and observed (×) concentrations at steady-state (*C*_*SS*_) of tamoxifen (TAM) **(A)** and endoxifen (ENDX) **(B)** dose-normalized and stratified by CYP2D6 phenotype categories and CYP-mediated coadministration as well as corresponding % non-attainment of therapeutic target endoxifen concentration **(C)**.

The visual predictive checks displayed that the simulated median, 25th-75th (dark shaded area) and 5th-95th (light shaded area) prediction intervals were in line with the observed concentrations over time after last dose at PK steady-state for tamoxifen and endoxifen (Supplementary Figure S2). Observed *C*_*SS,min*_ around 24 h were well captured by the model-predicted *C*_*SS,min*_ across subgroups and doses, as highlighted by the gray area in the VPC plots. The visual predictive box-whisker plots (Figure 7) revealed that the joint tamoxifen and endoxifen PK model reflected the observed *C*_*ss*_ data well when stratified by the four ‘traditional’ CYP2D6 phenotype categories (patients with AS = 1 classified as gNM) without and with CYP-mediated comedication intake, i.e., CYP3A-inducer rifampicin and CYP2D6-inhibiting SSRIs (Figures 7A,B). A slight overprediction of *C*_*SS,min ENDX*_ with co-administered rifampicin within the CYP2D6 gNM subgroup was apparent. An additional subdivision of the four ‘traditional’ CYP2D6 phenotype categories into the seven CYP2D6 AS subgroups revealed a good predictive performance as displayed in the VPC boxplots (see Supplementary Figure S3).

### Reduction of Unexplained Variability of Several Levels

Several levels of variability were dissected and precisely quantified by sequentially building up the hierarchical variability structure from two to four random variability levels (RUV, IIV, IOV, and ISV, see Table 1). IIV, IOV, and ISV were partly explained by the investigated covariates. By excluding covariate effects one by one from the full covariate model including 4-levels of variability, absolute and relative fractions of variability explained by the respective covariates were determined:

#### Interoccasion Variability

**FIGURE 8:**
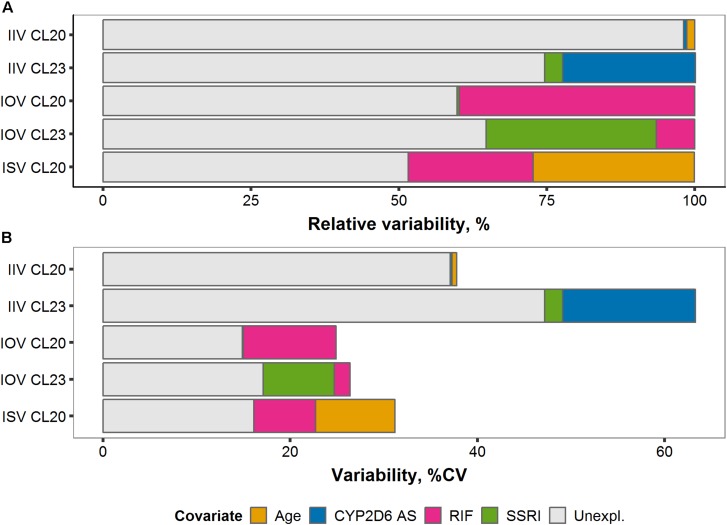
**(A)** Relative and **(B)** absolute fractions of variability (% coefficient of variation) explained by covariates on tamoxifen (TAM) clearance *CL_20_/F* and endoxifen (ENDX) formation *CL_23_/F* across the three levels of variability: Interindividual (IIV), interoccasion (IOV), and interstudy (ISV).

Large fractions, 39.8% and 28.8%, of the total IOV in CL20/F and CL23/F, respectively, were attributed to the introduction of the time-varying comedication covariate effects, rifampicin and SSRIs (Figure 8A). Once the effect of the CYP3A inducer rifampicin was implemented on CL20/F, the corresponding IOV reduced from 24.9% CV to 14.9% CV (Figure 8B). Similarly, considering the effect of coadministered potent CYP2D6 inhibitors SSRIs on CL23/F reduced the corresponding IOV from 26.4% CV to 17.1% CV.

#### Interindividual Variability

Of the total IIV in CL23/F, 25% (22.5% and 3.01%) were explained by the inclusion of the covariates CYP2D6 AS and SSRI comedication, respectively (Figure 8A), reducing the IIV of CL23/F by 35.2% from 63.3% CV to 47.2% CV (Figure 8B). A small fraction (1.35% and 0.54%) of total IIV in CL20/F was explained by the covariates age and CYP2D6 phenotype (Figure 8A), respectively.

#### Interstudy Variability

Upon covariate introduction, the total ISV reduced by more than half (51.8%) from 31.2% CV to 16.1% CV. Specifically, the addition of the covariate effects of age and rifampicin comedication reduced the total ISV on tamoxifen clearance (CL20/F) by 27.2% and 21.2%, respectively (Figure 8A).

Overall, large fractions of unexplained variability in IOV and ISV were explained upon covariate introduction resulting in CV values <18%. Although covariates explained considerable parts of IIV in CL23/F, IIVs on tamoxifen clearance (CL20/F) and endoxifen formation (CL23/F) remained large with CV of 37.1% and 47.2%, respectively.

### Determination of Patients at Risk of Subtarget Endoxifen Concentrations

The overall risk of subtarget endoxifen concentrations was similar in the observed (28.9%) and the simulated (27.1%) population, respectively. To identify subgroups at highest risk, the observed and simulated patient populations were stratified according to comedication intake and CYP2D6 AS. Predicted endoxifen concentrations at steady-state declined with decreasing CYP2D6 enzyme activity (from CYP2D6 ultrarapid, gUM, to poor metabolizers, gPM) and upon CYP-mediated comedication intake (CYP3A-inducer rifampicin and CYP2D6-inhibiting SSRIs) mimicking the overlaid observed data (Figure 7B).

CYP2D6 poor metabolizer (gPM), patients on rifampicin or on respective SSRIs were identified as subgroups at high risk of subtarget endoxifen concentrations, as indicated by the fractions predominately below the therapeutic threshold (dashed line in Figure 6, middle panel). For these subgroups, risk values were >76% with predicted risk values being well in line with the observed values in the PK database (Figure 7C).

CYP2D6 normal (gNM) and ultrarapid metabolizers (gUM) were at lower risk with 19.0% (95% CI: 15.3–23.5) and 0.0% (95% CI: 0.0–16.7), respectively. However, almost half of CYP2D6 intermediate metabolizers (gIM) were at risk of subtarget endoxifen concentrations with 42.3% (95% CI: 26.9–59.6). gPM and patients that co-administered CYP2D6-inhibiting SSRIs (paroxetine/fluoxetine) revealed similar risk values with 75.6% (95% CI: 57.8–88.9) and 76.5% (95% CI: 55.9–91.2), respectively. 100% risk of subtarget endoxifen concentrations were displayed in gIM and gPM that co-administered CYP2D6-inhibiting SSRIs, as well as in patients co-administering rifampicin independent of their CYP2D6 phenotype.

### Standard vs. Two More Individualized Tamoxifen Dosing Strategies

**FIGURE 9:**
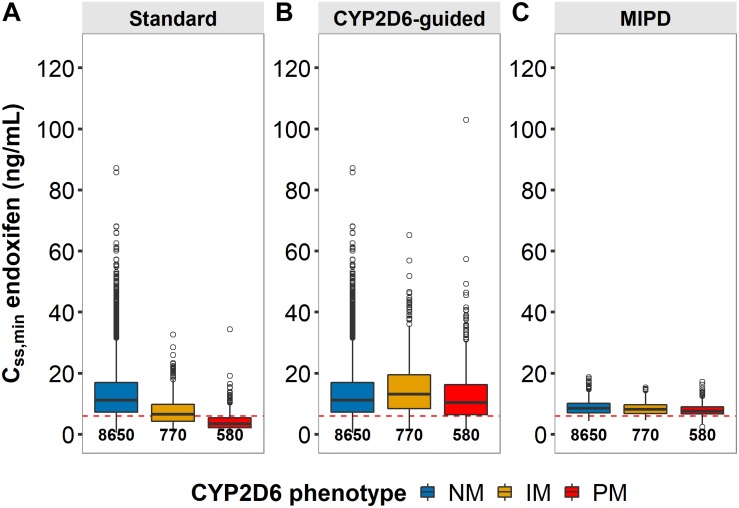
Patients at risk of subtarget endoxifen concentrations across dosing strategies. Simulated minimum steady-state concentrations of endoxifen (*C*_*SS,min*_) in CYP2D6 genotype-predicted normal/ultrarapid metabolizers (gNM), intermediate (gIM) and poor metabolizer (gPM) across dosing strategies **(A)** standard, **(B)** CYP2D6-guided and **(C)** model-informed precision dosing (MIPD) (*n*_*each*_ = 10,000). *Dashed horizontal line:* endoxifen therapeutic threshold (Madlensky et al., 2011); *boxes*: interquartile range (IQR), including median; *whiskers:* range from hinge to lowest/highest value within 1.5 IQR; *points*: data outside whiskers.

**FIGURE 10:**
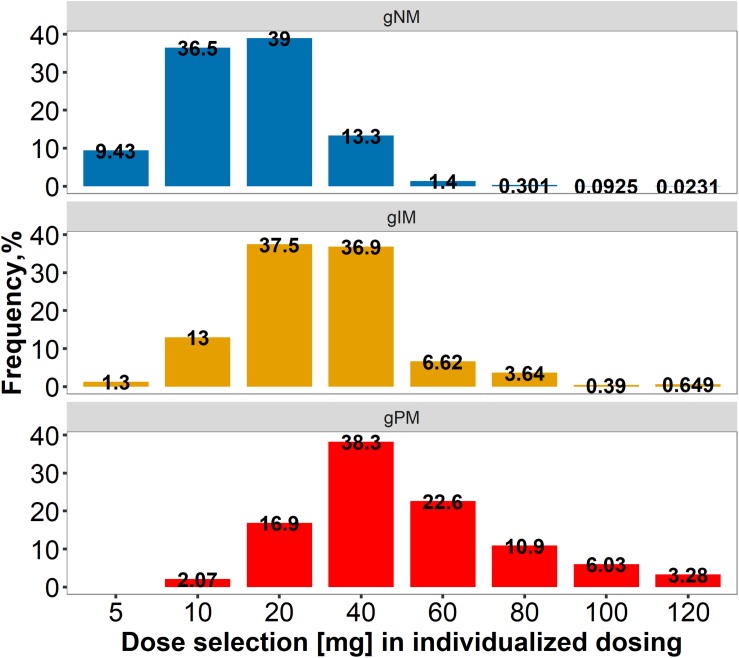
Dose spread within and across CYP2D6 normal/ultrarapid, intermediate and poor metabolizers, applying model-informed precision dosing. *gPM/gIM/gNM/*: poor/intermediate/normal incl. ultrarapid metabolizer, predicted from genotype.

Three dosing strategies were compared using a simulation approach applying the previously developed joint parent-metabolite pharmacokinetic model. In the standard dosing group (strategy 1), 8 out of 10 gPM (80.2%) and almost half (45.2%) of gIM were at risk of subtarget endoxifen concentrations (Figure 9A). Overall, 22.2% of the virtual population were at risk in the standard dosing group. For CYP2D6-guided dosing (strategy 2), daily doses of 40 mg in gIM and 60 mg in gPM were appropriate to reduce risks for subtarget endoxifen concentrations to 10.4% and 20.3%, respectively, and to obtain similar endoxifen concentrations as observed in gNM at 20 mg daily doses (Figure 9B). However, a large variability in *C*_*SS,min ENDX*_ within the CYP2D6 sub-populations was observed (%CV > 59%, see Figure 9B and Table 3). Thus, CYP2D6-guided dosing resulted in very high concentrations of tamoxifen and its metabolites in some patients. MIPD (strategy 3) facilitated target attainment in almost all patients. Concretely, the risk was reduced to 6.88% in gNM, 7.67% in gIM and 11.0% in gPM. Moreover, a narrow range in *C*_*SS,min ENDX*_ was achieved within and across all subpopulations (Figure 9C and Table 3). As a consequence, a large dose spread was obtained (see Figure 10): while for the majority of gIM and gPM, increased doses of up to 40 mg or 60 mg QD were sufficient to attain therapeutic endoxifen concentrations, the majority of gNM were adequately treated with the standard dose. Only in rare cases, gPM required high doses up to 120 mg QD. Of note, the PK target threshold was achieved with lower doses than the CYP2D6-guided dose for a considerable number of gNM (5 mg or 10 mg: 45.8%), gIM (<40 mg: 51.8%) and gPM (<60 mg: 57.2%) when compared with dosing strategy 2. Most importantly, in total 92.1% of patients achieved therapeutic *C*_*SS,minENDX*_ in the MIPD group whereas it was only 84.0% in CYP2D6-guided dosing and 77.8% in standard dosing.

**TABLE 3:** Comparison of clinical trial simulations of standard vs. CYP2D6-guided- vs. model-informed precision dosing.

	**(1) Standard dosing**				**(2) CYP2D6-guided dosing**				**(3) Model-informed precision dosing**			
CYP2D6	gNM	gIM	gPM	All	gNM	gIM	gPM	All	gNM	gIM	gPM	All
*n*_patients_	8,650	770	580	10,000	8,650	770	580	10,000	8,650	770	580	10,000
Dose (mg)^a^	20 (−)	20 (−)	20 (−)		20 (−)	40 (−)	60 (−)		20 (5–120)	40 (5–120)	60 (10–120)	
*C*_SS,minENDX_												
−Median	12.4	6.54	3.44		12.4	16.4	10.3		8.49	8.37	7.55	
−IQR	7.24–16.7	4.22−9.73	2.16−5.42		7.24−16.7	10.9−24.4	6.48−16.3		7.06–10.2	6.99–10.0	6.64–9.00	
−CV,%	63.8	59.0	72.3		61.1	64.9	72.3		23.9	23.9	24.9	
*n*_*risk*, %_	16.2	45.2	80.2	22.2	16.2	10.4	20.3	16.0	6.88	7.67	11.0	7.19

**For all three scenarios, the same virtual tamoxifen population (n = 10,000) was used. a: fixed or median dose (range); allowed dose range 5–120 mg (min-max); C_*SS,minENDX*_: minimum endoxifen steady-state concentration in ng/mL; CV: coefficient of variation assuming log-normal distributed C_*SS,minENDX*_; all: total population if respective scenario; IQR: inter-quartile range; gNM/gIM/gPM: normal incl. ultrarapid/intermediate/poor metabolizer; n_*risk*_: patients at risk of subtarget endoxifen concentrations; PTA: probability of endoxifen PK target-attainment.**

## Discussion

A parent-metabolite PK model was successfully developed, jointly describing tamoxifen and its clinically most important metabolite endoxifen, following continuous once daily oral tamoxifen dosing. The integrated analysis of six pooled clinical studies identified a patient’s CYP2D6 genotype as predictor for *C*_*SS,minENDX*_ and as a highly informative covariate for potential dose adjustments at tamoxifen treatment initiation. Furthermore, drug–drug interactions with strong CYP2D6 inhibitors or strong CYP3A4 inducers were shown to dramatically reduce endoxifen concentrations, which might impede tamoxifen treatment success and should thus be evaluated in a dedicated prospective clinical trial. Most importantly, the low observed IOV in contrast to the high observed IIV demonstrates the potential of individualized dosing for treatment optimization. To provide useful guidance in clinical practice on the one hand, and a potentially more successful treatment for the individual tamoxifen patient on the other hand, a model-informed precision dosing strategy as demonstrated by the investigated simulation study might be most appropriate.

The CYP2D6 AS was identified as a strong predictor of endoxifen exposure, as its impact on endoxifen concentrations was precisely estimated and large, relevant effect sizes were displayed (Figure 6). Importantly, the model successfully predicted increasing CL23/F with increasing CYP2D6 AS and corresponding percentage risk values across the full range of CYP2D6 activity scores (only lumping AS groups of 2.5 and 3) (Supplementary Figure S2). With almost all patients displaying subtarget endoxifen concentrations, gPM (AS: 0) were identified as patients at highest risk, followed by gIM (AS: 0.5) with around half of patients at risk. These findings are consistent with previous findings in the literature (Borges et al., 2006; Madlensky et al., 2011; Mürdter et al., 2011; Schroth et al., 2017) and substantiate the important role of CYP2D6 in tamoxifen pharmacokinetics. As patients’ CYP2D6 genotype is easily determined or already accessible prior to treatment start, it should be considered for initial dose selection. Neglecting this information might cause several additional and unnecessary steps of dose refinements until the endoxifen target concentration is met.

Integrated data from patients comedicated with rifampicin or SSRIs revealed substantial alterations on the PK of tamoxifen and endoxifen. Most remarkably, *C*_*SS,min ENDX*_ fell below the therapeutic threshold during rifampicin coadministration, irrespective of CYP2D6 phenotype (Figure 8). During strong CYP2D6-inhibitor comedication, the inhibition of endoxifen formation was substantial (−65%) resulting in gNM showing FM comparable to CYP2D6 gPM (2.32%) and gNM coadministering rifampicin (2.42%). This was not surprising, since our observations were well in line with reports from literature (Stearns et al., 2003; Jin et al., 2005; Borges et al., 2006). These findings call for action to reduce the risk of CYP-mediated DDIs in tamoxifen therapy, as they may hamper clinical efficacy (Hansten, 2018).

While all identified covariates have been described before, we for the first time evaluated and quantified their impact jointly in a large real-world patient cohort. In addition, our model-based analysis enabled to determine and precisely estimate for each covariate the specific mechanism influenced (i.e., endoxifen formation by CYP2D6 AS and tamoxifen clearance by rifampicin coadministration).

A particular strength of our study was the large and diverse tamoxifen population which enabled to dissect and quantitatively characterize the PK variability of tamoxifen and endoxifen across studies (i.e., ISV), between (i.e., IIV) and within patients (i.e., IOV and RUV). As expected, large fractions of IOV were explained by the time-varying covariates (rifampicin and SSRI comedication) resulting in a low IOV in the final NLME-PK model (≤17.1% CV). Thus, within a patient, plasma concentrations of tamoxifen and endoxifen were relatively constant over time and therefore predictable for future treatment. In contrast, large unexplained IIV in the PK of tamoxifen and endoxifen, as reflected by the large IIV values on tamoxifen elimination (39.9% CV) and endoxifen formation (47.2% CV), was observed. These individual differences in PK may lead to differences in clinical response. Hence, to ensure therapeutic *C*_*SS,minENDX*_ in the individual patient [applying the proposed threshold by Madlensky et al. (2011)], a dosing strategy that considers a patient’s individual PK might be most promising. Model-informed precision dosing facilitates the systematic and quantitative consideration of various influential factors on the PK of tamoxifen, i.e., CYP2D6 functional activity, patient age and comedication, in the individual dose selection process. A low IOV [<20% (Abrantes et al., 2019)] and a high IIV enable to predict drug concentrations upon potential dose adaptations (Karlsson and Sheiner, 1993; Wallin et al., 2010; Abrantes et al., 2019) and are thus important predictors for added value by MIPD, as it was demonstrated in our simulation study.

In this simulation study, the currently practiced ‘one-dose-fits-all’-strategy, in which all patients receive 20 mg tamoxifen daily irrespective of their CYP2D6 AS, performed poor with respect to both (i) endoxifen target attainment and (ii) the large interindividual variability of *C*_*SS,minENDX*_. Both alternative strategies, CYP2D6-guided dosing and MIPD, were superior in scope of the probability of target attainment. Applying MIPD reduced the risk for subtarget *C*_*SS,minENDX*_ by 2/3 compared to standard dosing. This corresponds to a number needed to treat (NNT) of 7, meaning that for one additional patient to reach target *C*_*SS,minENDX*_, only 7 patients would have to be treated applying MIPD instead of standard dosing. While MIPD was most beneficial for gIM and gPM (NNT of 3 and 2, respectively), there was also a substantial effect for gNM (NNT: 11). While the CYP2D6 AS can guide initial tamoxifen treatment as predictive covariate, TDM samples after 2, 3, and 4 weeks of CYP2D6-guided tamoxifen treatment may further individualize the optimal tamoxifen dose. Furthermore, MIPD results in a narrow *C*_*SS,minENDX*_ range even among patients with diverse CYP2D6 functionality, due to the wide spread of individually selected doses.

TDM-supported dosing without modeling and simulation may be considered as more intuitive and more versatile, but we have to consider that without modeling and simulation in general (i) steady-state has to be attained first and (ii) TDM samples have to be taken at exact time points to be reliable. We have previously reported that fluctuations of *C*_*SS,minENDX*_ within a dosing interval are minimal (Klopp-Schulze et al., 2018), which in this special case allows to take TDM samples at any time during a steady-state dosing interval. Nevertheless, in TDM ‘only’ it is generally crucial to obtain samples at predefined timepoints (mostly *C*_*min*_) to be able to compare measured with target concentrations. In contrast, deviations from scheduled sampling times are unproblematic in MIPD as long as exact sampling timepoints are documented. Even though TDM-supported tamoxifen dosing without modeling and simulation is feasible with respect to sampling timepoints, it takes about 1 and 3 months, respectively, for tamoxifen and endoxifen, to reach steady-state and a considerable proportion of patients will need several months to reach target endoxifen steady-state concentrations. Furthermore, dose adaptations based on TDM ‘only’ are more sensitive to data errors and thus only precise if multiple samples per patient are available. Calculations of PK target indices to derive dose recommendations from TDM samples take additional time, efforts and clinical staff with appropriate skills to perform the analysis (using adequate software programs). Finally, the interpretation of TDM results and translation into a dose recommendation requires specific knowledge and thereby depends on the attending clinician/clinical pharmacist. MIPD, in contrast, not only protects against measurement errors and process noise but also enables to incorporate relevant predictors, i.e., genetic information, and requires fewer samples by considering optimal sampling time windows (‘optimal design’) (D’Argenio, 1981). By combining prior knowledge (integrated in the model), patient characteristics (available before treatment start) and patient individual PK information (from TDM samples), therapeutic concentrations can be achieved earlier and with higher probability, compared to standard or TDM-based dosing strategies (Dezentjé et al., 2015; Fox et al., 2016). The feasibility of our proposed MIPD framework might be challenged as we propose doses as low as 5 mg and as high as 120 mg, both requiring off-label use of tamoxifen. Even though we made sure to only consider off-label doses which had been tested and proven safe in clinical studies (Dezentjé et al., 2015; DeCensi et al., 2019), additional information on the efficacy and safety of off-label tamoxifen doses has to be generated in clinical trials before their use can be recommended in clinical practice.

Of note, tamoxifen’s complex PK is ascribed to its physicochemical properties but also causes challenges from a bioanalytical perspective. More than one decade after the discovery of the active metabolite endoxifen, standardized validated bioanalytical methods have yet to be established to assure comparable PK and PGx results (Gaedigk et al., 2008; Brauch et al., 2013; Ratain et al., 2013; Schroth et al., 2017). This probably plays not only a role for the characterization of IIV but also of ISV. These – yet undetermined – interstudy discrepancies have also been identified when comparing predictions from two published NLME-PK models of tamoxifen and endoxifen (Klopp-Schulze et al., 2015, 2018). These PK models characterized the PK of the underlying clinical data, which largely differed in patient size, distribution of un-/documented patient factors, study design and bioanalytical assessment. These aspects are important to be aware of and need to be carefully considered when re-purposing a PK model based on one dataset, e.g., for simulations or model-informed dosing. To obtain a more complete representation of the ‘real-world’ patient population in clinical routine, it is appealing to integrate clinical data from multiple studies into a single NLME-PK model (meta-analysis approach), as has been done in this work.

Importantly, it should be considered that interindividual variability in clinical outcome might not only be explainable by variability in PK but also by variations in pharmacodynamics. The latter contributes to the difficulties observed in reproducing and confirming the relationship between endoxifen exposure and clinical outcome. Therefore, carefully designed prospective studies are needed, jointly assessing PK and clinical endpoints (breast cancer progression/recurrence, adverse drug reactions) and/or an appropriate biomarker [e.g., tumor size, circulating tumor cells, Ki67 antigen (Yerushalmi et al., 2010) over time (Ribba et al., 2014; Bender et al., 2015; Buil-Bruna et al., 2016)] in a representative, well-defined tamoxifen patient population. In this respect and as demonstrated in the presented simulation studies, modeling and simulation provides the potential to elucidate not only the complex PK behavior of tamoxifen and its metabolites, but also to assess the drug and/or metabolites exposure-response relationship.

Of note, endoxifen as own compound is currently in clinical development (Ahmad et al., 2010a, b; Goetz et al., 2017) and could supersede tamoxifen as antihormonal breast cancer drug in the future. Given the independence of endoxifen formation by CYP2D6 and the opportunity to bypass the complex metabolism of tamoxifen, this development seems straightforward and promising. However, the relevance of tamoxifen itself and its other metabolites for its efficacy has still not been fully elucidated. As a first step, the prospective, multicentre TAMENDOX study (NCT03931928) (TAMENDOX, 2020) will assess the feasibility of genotype- and phenotype-guided supplementation of tamoxifen standard therapy with endoxifen to reach target *C*_*SS,minENDX*_. Future clinical studies are warranted to show if endoxifen is superior to model-informed precision dosed tamoxifen with respect to clinical outcomes.

Despite the large potential of MIPD, it is important to mention that the relationship between CYP2D6 genotype and/or endoxifen concentrations and breast cancer recurrence is still controversial and has been the subject of intensive debates amongst researchers and clinicians since almost a decade (Brauch et al., 2013; Binkhorst et al., 2015b; Zembutsu et al., 2017; Neven et al., 2018). A recent study by Sanchez-Spitman et al. (2019), in which no relationship between CYP2D6 genotype or endoxifen concentrations and clinical outcome could be established, revived the discussion once again. Several researchers criticized large methodological shortcomings of this work (Braal et al., 2019; Brauch et al., 2019; Goetz et al., 2019), i.e., low power of only ∼30% to detect the proposed exposure-response relationship, lack of information on co-medication, and limited/inadequate PK sampling (de Vries Schultink, 2019). A common element of all responses was the urgent call for a well-designed prospective, randomized clinical trial with sufficient power to investigate the proposed exposure-response relationship. Simulations of observational and randomized clinical trials to evaluate the feasibility of TDM for endoxifen showed that at least low thousands of patients would have to be followed for 15 years or longer in observational or randomized controlled trials, respectively, for sufficient power (>0.8) to confirm the hazard ratio of 0.71 (de Vries Schultink, 2019) reported by Madlensky et al. (2011) and in line with Saladores et al. (2015) and Gong et al. (2013).

Based on the still considerable controversy over the relationship between CYP2D6 genotype and/or endoxifen concentrations and clinical outcome, our theoretical *in silico* simulation work suggests as next step the investigation of our proposed MIPD framework in comparison to the conventional ‘one-dose-fits-all’ dosing strategy in a well-designed and -powered prospective clinical trial setting, allowing to evaluate the ‘real-world’ clinical benefit (i.e., the possibility of achieving recduced breast cancer recurrence rates) and (long-term) safety of MIPD for tamoxifen. Furthermore, as treatment outcome cannot be explained by endoxifen concentrations alone, other relevant factors contributing to the overall variability in clinical outcome need to be identified and appropriately characterized.

In summary, this presented model provides an appropriate framework to (i) identify patients at risk of subtarget endoxifen concentrations prior to tamoxifen treatment start, (ii) aid an upfront dose adaptation based on a patient’s CYP2D6 geno-/phenotype, and (iii) guide dose refinements early after treatment start based on TDM samples, as well as (iv) monitor and detect discrepancies, due to, e.g., non-adherence or DDIs, allowing enhanced patient care and improved clinical benefits.

The outlined modeling and simulation framework might be well translatable and applicable to further oral anti-anticancer drugs (OADs) exhibiting similar challenges. A model-informed precision dosing tool for a collection of OADs could conceivably be of high utility to aid rational dose decision-making in routine oncology clinical practice, e.g., by expanding existing model-based dose decision support tools [such as TDMx (Wicha et al., 2015) or InsightRx (InsightRx, 2019)] to the therapeutic area of oncology.

## Data Availability Statement

The datasets for this manuscript are not publicly available because patients did not provide consent for their data being shared in a public database and this purpose was also not included in the original IRB proposal. Requests to access the datasets should be directed to the corresponding author.

## Ethics Statement

The studies involving human participants were reviewed and approved by the respective ethics committees as this is a pooled meta-analysis of original studies. The patients/participants provided their written informed consent to participate in this study.

## Author Contributions

LK-S, AM-S, MJ, and CK contributed conception and design of the presented work and discussed the results. LK-S created the database. LK-S and AM-S performed the modeling and simulation analyses. LK-S and AM-S wrote the first draft of the manuscript. MJ, PN, RM, and SK contributed to sections of the manuscript. All authors contributed to manuscript revision, read and approved the submitted version.

## Conflict of Interest

LK-S is currently employed by Merck Healthcare KGaA, Germany. CK reports grants from an industry consortium (AbbVie Deutschland GmbH & Co. KG, Boehringer Ingelheim Pharma GmbH & Co. KG, Grünenthal GmbH, F. Hoffmann-La Roche Ltd., Merck KGaA and Sanofi) for the PharMetrX program and the Innovative Medicines Initiative-Joint Undertaking (‘DDMoRe’).

The remaining authors declare that the research was conducted in the absence of any commercial or financial relationships that could be construed as a potential conflict of interest.
