# Supplementary material for: Integrated Data Analysis of Six Clinical Studies Points Toward Model-Informed Precision Dosing of Tamoxifen
Source: Front Pharmacol. 2020 Mar 31;11:283. doi: 10.3389/fphar.2020.00283 (PMC7136483; doi:10.3389/fphar.2020.00283)
Supplement: Supplementary file 1 [file Data_Sheet_1.docx]

Supplementary Material

# Supplementary Data

- 1. **Extended information on the six clinical tamoxifen studies featured in the clinical PK database**

**Study 1: An Observational Study to Assess Response to Tamoxifen in Breast Cancer Patients (CYPTAMBRUT-2)** (1)

**Treatment setting:** Neo-adjuvant, primary metastatic, metastatic
**Prior tamoxifen use:** No
**Analytical method:** Validated HPLC LC/MS/MS method using serum

| **Inclusion criteria** | **Exclusion criteria** |
| --- | --- |
| - Female>18 years of age - Written and voluntary informed consent understood signed and dated - Histologically or cytologically confirmed measurable invasive adenocarcinoma of the breast either large (cT3), locally advanced stage IIIB/C inoperable, or metastatic and not amenable to curative therapy with surgery or radiotherapy - Measurable disease is defined as follows: CT scan for metastatic or locally advanced stage IIIb disease and ultrasound of the breast for operable large size breast cancers where tamoxifen is given for neoadjuvant endocrine therapy - Patients must be postmenopausal. - Breast cancer should be considered as oestrogen receptor positive by the clinician using immunohistochemistry readings as is standard procedure for local pathologist. - Prior endocrine tamoxifen therapy in the adjuvant setting is allowed if there is more than 12 months after completion of adjuvant tamoxifen - Prior radiotherapy is allowed but evaluable lesions that have been irradiated need to be progressive before starting in the study - Concurrent use of bisphosphonates is allowed if they are started 2 weeks before study start and these drugs should be continued as planned throughout the study - Adequate renal and liver function Serum creatinine and serum bilirubin ≤1.5xULN, serum ALT and AST ≤2.5x ULN (or ≤5 in case of liver metastases) - Serum calcium should be ≤11.6 mg/dL - ECOG performance status: 0-2 | - Male - Life threatening disease requiring a quick response (e.g. extensive hepatic or pulmonary involvement) - CNS involvement - Less than 12 months since stopping tamoxifen in the adjuvant setting - Previous chemotherapy, tamoxifen or more than one line of hormonal therapy or targeted therapy for locally advanced/metastatic breast cancer - Bone lesions only - One line of prior endocrine therapy with an oral aromatase inhibitor for locally advanced or metastatic breast cancer is not allowed also not if there is clear progression according to RECIST and the clinician judges tamoxifen an appropriate second line therapy - Contraindication for tamoxifen: history of DVT/vaginal bleeding of unknown origin - Dementia - History of other malignancy that may interfere with at least 6 months of tamoxifen therapy |

**Study 2: Prevalence of Genetic Polymorphisms in Gene Coding for Tamoxifen Metabolising Enzymes (CYPTAMBRUT-3)** (2)

**Treatment setting:** Adjuvant
**Prior tamoxifen use:** No
**Analytical method:** Validated HPLC LC/MS/MS method using serum

| **Inclusion criteria** | **Exclusion criteria** |
| --- | --- |
| - Female>18 years of age - Written and voluntary informed consent understood signed and dated - Histologically or cytologically confirmed measurable invasive adenocarcinoma of the breast, amenable to curative therapy - Patients must be postmenopausal - Breast cancer should be considered as oestrogen receptor positive by the clinician using immunohistochemistry readings as is standard procedure for local pathologist. - Prior endocrine tamoxifen therapy is not allowed - Patients are not previously treated with an endocrine agent or hormone replacement therapy needs being stopped for at least 6 months - Prior chemotherapy and radiotherapy is allowed - Adequate renal and liver function Serum creatinine and serum bilirubin ≤1.5xULN, serum ALT and AST ≤2.5x ULN (or ≤5 in case of liver metastates) - Serum calcium should be ≤11.6 mg/dL - ECOG performance status: 0-2 | - Male - Life threatening disease requiring a quick response (e.g. extensive hepatic or pulmonary involvement) - Use of any endocrine treatment or recent/current use of hormone replacement therapy - Contraindication for tamoxifen: history of DVT/vaginal bleeding of unknown origin - Dementia - History of other malignancy that may interfere with at least 6 months of tamoxifen therapy |

**Study 3: Studying the relationship between the CYP3A and CYP2D6 probe dextromethorphan and the pharmacokinetics of tamoxifen** (3)

**Treatment setting:** Adjuvant or metastatic
**Prior tamoxifen use:** ≥3 weeks
**Analytical method:** Validated HPLC LC/MS/MS method using serum (4)

| **Inclusion criteria** | **Exclusion criteria** |
| --- | --- |
| - Histological or cytological confirmed diagnosis of breast cancer, for which treatment with tamoxifen is indicated - Use of tamoxifen for at least 4 weeks (to guarantee steady-state) - Age ≥18 years - WHO performance ≤1 - Adequate hematological blood counts - Written informed consent - Adequate renal and hepatic functions - Use of tamoxifen monotherapy for at least 3 weeks | - Pregnant or lactating patients - Patients with reproductive potential must use a reliable method of contraception - Impossibility to take oral drugs - Serious illness or medical unstable condition requiring treatment - Symptomatic CNS-metastases or history of psychiatric disorder that would prohibit the understanding and giving of informed consent - Unwillingness to abstain from grapefruit (juice), (herbal) dietary supplements, herbals and over the counter medication (except paracetamol and ibuprofen) and other drugs known to seriously interact with CYP3A and/or ABCB1 and/or ABCG2 during the study period - Use of strong CYP3A and/or P-glycoprotein inhibiting and inducing medication dietary supplements or other inhibiting compounds |

**Study 4: Optimizing endoxifen concentration through the induction of CYP3A4, CYP2C and CYP2D6 mediated tamoxifen metabolism** (5)

**Treatment setting:** Adjuvant
**Prior tamoxifen use:** ≥4 weeks
**Analytical method:** Validated UPLC MS/MS assay using plasma (6)

| **Inclusion criteria** | **Exclusion criteria** |
| --- | --- |
| - Histological or cytological confirmed diagnosis of breast cancer, for which treatment with tamoxifen monotherapy is indicated - Use of tamoxifen for at least 4 weeks (to guarantee steady-state) and willing to continue the treatment until the end of the study - Age >18 years - WHO performance <1 - Adequate hematological blood counts - Written informed consent - Adequate renal and hepatic functions - No chemotherapy or radiotherapy within the last 4 weeks before start - No concurrent (over the counter) medication or (herbal) supplements known to induce or inhibit CYP2D6, CYP2C, CYP3A4 and/or P-glycoprotein - No concurrent medication or supplements which can interact with rifampicin - Abstain from grapefruit, grapefruit juice, herbal dietary supplements and herbal tea during the study | - Pregnant or lactating patients - Impossibility to take oral drugs - Serious illness or medical unstable condition requiring treatment, symptomatic CNS metastases or history of psychiatric disorder that would prohibit the understanding and giving of informed consent - Contraindications for rifampicin and/or dextromethorphan use - Use of medications or dietary supplements known to induce or inhibit CYP2D6, CYP2C, CY3A5 and/or P-glycoprotein - Unwillingness to abstain from grapefruit (juice), (herbal) dietary supplements, herbals, over-the-counter medication (except for low dose of paracetamol and ibuprofen) and other drugs known to seriously interact with CYP3A during the study period - More than one tamoxifen dose per day (20 or 40 mg) - Non-compliance |

**Study 5: The effects of switching antidepressants on endoxifen exposure** (7) **Treatment setting:** Adjuvant
**Prior tamoxifen use:** ≥4 weeks
**Analytical method:** Validated UPLC MS/MS assay using plasma (6)

| **Inclusion criteria** | **Exclusion criteria** |
| --- | --- |
| - Histological or cytological confirmed diagnosis of breast cancer, for which treatment with tamoxifen is indicated - Use of tamoxifen for at least 4 weeks (to guarantee steady-state) - Age >18 years - WHO performance <1 - Adequate hematological blood counts - Written informed consent - Adequate renal and hepatic functions - No chemotherapy or radiotherapy within the last 4 weeks before start - No concurrent (over the counter) medication or (herbal) supplements, except SSRIs, known to induce or inhibit CYP2D6, CYP2C, CYP3A4 and/or P-glycoprotein - No concurrent medication or supplements which can interact with venlafaxine and/or escitalopram - Abstain from grapefruit, grapefruit juice, herbal dietary supplements and herbal tea during the study | - Pregnant or lactating patients - Serious illness or medical unstable condition requiring treatment, symptomatic CNS metastases or history of psychiatric disorder that would prohibit the understanding and giving of informed consent - Patients with a history of suicide attempts or current suicidal ideation - Contraindications for venlafaxine and/or escitalopram use - Patients with Congenital Long QT Syndrome (CLQTS) - Use of medications or dietary supplements, except SSRIs, known to induce or inhibit CYP2D6, CYP2C, CY3A5 and/or P-glycoprotein - More than one tamoxifen dose per day (20 or 40 mg) - Non-compliance |

**Study 6: The influence of morning versus evening administration on tamoxifen pharmacokinetics** (8) **Treatment setting:** Adjuvant and metastatic
**Prior tamoxifen use:** ≥4 months
**Analytical method:** Validated UPLC MS/MS assay using plasma (6)

| **Inclusion criteria** | **Exclusion criteria** |
| --- | --- |
| - Histological or cytological confirmed diagnosis of breast cancer, for which treatment with tamoxifen is indicated - Use of tamoxifen for at least 4 weeks and willing to continue the treatment until the end of the study - Age >18 years - WHO performance <1 - Written informed consent - Adequate renal and hepatic functions - Adequate hematological blood counts - No chemotherapy or radiotherapy within the last 4 weeks before start | - Pregnant or lactating patients - Serious illness or medical unstable condition requiring treatment, symptomatic CNS metastases or history of psychiatric disorder that would prohibit the understanding and giving of informed consent - More than one tamoxifen dose per day (20 or 40 mg) - Non-compliance |

## 1.2. Covariate submodel development

**Step 1: Covariate pre-selection** The pre-selection of covariate-parameter relationships was based on the following criteria: (a) Prior knowledge from literature, pharmacokinetic and -genetic information of tamoxifen and endoxifen, especially from previous (physiologically-based) pharmacokinetic ((PB)PK) models (9–11) (b) mechanistic/biological plausibility, (c) context of use, i.e. clinical utility in terms of model-informed (therapeutic drug monitoring (TDM)) dosing, (d) scientific interest and (e) sufficient information content in PK database to support analysis (< 20% missing covariate values). In addition, (f) exploratory statistical and graphical analyses were used to guide the covariate model pre-selection, to generate expectations on the covariate effect size and on potential model functions by examining C_SS,min_ versus covariate and covariates versus interindividual variability (IIV) plots (e.g. see Figure 4 in main text).

**Step 2: Covariate model refinement** To determine the most appropriate covariate-parameter relationships for covariates age and CYP2D6, statistical significance test (LRT), parameter precision, model stability and physiological plausibility were used to guide the covariate model refinement procedure.

**Step 3: Final covariate model selection and evaluation** To justify the final covariate selection, physiological plausibility, parameter precision, reduction of unexplained variability, clinical relevance and statistical significance were jointly examined taking into account the model purpose and its clinical utility. In addition, goodness-of-fit plots, e.g. of covariates versus IIV were inspected for remaining trends. The resulting joint tamoxifen-endoxifen PK model was finally evaluated using advanced diagnostic techniques (see Section 1.2 below).

### 1.2.1 Covariate model: Comedication

Rifampicin and SSRI comedication were introduced as dichotomous covariates (0: not taken, i.e. reference category; 1: taken) into the model. For both, the covariate-parameter relationship was characterised using a fractional change model (Equation 1).

${CL}_{i}=\theta_{Ref}\cdot\left( 1+\theta_{Comed}\cdot X_{i} \right)\cdot e^{\eta_{i,CL}}$ (Eq. 1)

In Equation 1, $\theta_{Ref}$ represents the clearance if no comedication was administered (indicator variable $X_{i}$= 0) and $\theta_{Comed}$ describes the fractional change in clearance if the comedication was taken switching $X_{i}$ to 1.While rifampicin comedication, as strong CYP3A inducer altering CYP3A pathways, was implemented on both parameters CL20/F and CL23/F, SSRI comedication, i.e. paroxetine and fluoxetine as potent CYP2D6 inhibitors influencing CYP2D6 pathways, was incorporated on CL23/F.

### 1.2.2 Covariate model refinement: Age and CYP2D6

Using the a priori-specified full covariate model as starting point (comedication was modelled as fractional change model, whereas age and CYP2D6 AS were introduced using linear models), several covariate functions of age and CYP2D6 on PK were investigated:

**Age** The influence of age on the tamoxifen clearance (CL20/F) was investigated using linear, exponential and power functions (Equation 2-4), normalised and/or centered by the reference age ${Age}_{Ref}$ of 65 years (i.e. median value of the tamoxifen PK database and commonly used as reference age in adults) (12,13)

$CL20/F_{i}=\theta_{Ref}\cdot\left( 1+\theta_{Age}\cdot\frac{{(Age}_{i}-{Age}_{Ref})}{{Age}_{Ref}} \right)\cdot e^{\eta_{i,CL20}}$ (Eq. 2)

$CL20/F_{i}=\theta_{Ref}\cdot e^{(\theta_{Age}\cdot{(Age}_{i}-{Age}_{Ref}))}\cdot e^{\eta_{i,CL20}}$ (Eq. 3)

$CL20/F_{i}=\theta_{Ref}\cdot\left( \frac{{Age}_{i}}{{Age}_{Ref}} \right)^{\theta_{Age}}\cdot e^{\eta_{i,CL20}}$ (Eq. 4)

In these models CL20/F represents θ_Ref_ (the typical tamoxifen clearance) when Age = 65 (i.e. reference age), and θ_Age_ describes the estimated effect of age on CL20/F.

**CYP2D6 activity score** The CYP2D6 AS is a multichotomous covariate with seven ordered categories from 0 to 3 (step size of 0.5). For each patient in the PK database, an AS was derived from the reported CYP2D6 genotype.

Two covariate modelling strategies for the CYP2D6 activity score were investigated with three different mathematical implementations each:

1. The AS was treated as continuous covariate investigating linear, power and exponential relationships on endoxifen formation (CL23/F) (Equation 5-7);

$CL23/F_{i}=\theta_{Ref}\cdot\left( 1+\theta_{AS}\cdot\frac{{(AS}_{i}-{AS}_{Ref})}{{AS}_{Ref}} \right)\cdot e^{\eta_{i,CL23}}$ (Eq. 5)

$CL23/F_{i}=\theta_{Ref}\cdot e^{(\theta_{AS}\cdot{(AS}_{i}-{AS}_{Ref}))}\cdot e^{\eta_{i,CL23}}$ (Eq. 6)

$CL23/F_{i}=\theta_{Ref}\cdot\left( \frac{{AS}_{i}+1}{{AS}_{Ref}+1} \right)^{\theta_{AS}}\cdot e^{\eta_{i,CL23}}$ (Eq. 7)

1. The AS was introduced as ordered categorical covariate applying a fractional change model with four categories representing the ‘traditional’ CYP2D6 phenotype classes investigating two different grouping types for the intermediate (IM) and normal (NM) metaboliser group (depending on whether AS=1 is classified as IM or NM, see Figure 1 in main text), or with six AS categories only grouping AS 2.5 and 3 (Equation 8). To model the CYP2D6 activity scores, fiveseparate categorical covariate effects (for AS subgroups: 0, 0.5, 1, 1.5 and 2.5-3) were estimated while AS=2 was set as reference category (i.e. for a typical patient with AS=2: CL23/F =$\theta_{Ref}$). An indicator variable $X_{AS,i}$ was coded as 1 for a patient belonging into the respective AS category or 0 otherwise.

$CL23/F_{i}=\theta_{Ref}\cdot\left( 1+\theta_{AS:0}\cdot X_{AS:0,i}+\ldots+ \theta_{AS:3}\cdot X_{AS:3,i} \right)\cdot e^{\eta_{i,CL23}}$ (Eq. 8)

Hence, depending on a patient’s CYP2D6 activity score and its corresponding indicator variable $X_{AS:x,i}$, CL23/F_i_ could take on the following values under the fractional change model (here exemplified for poor, gPM, and ultrarapid metabolisers, gUM, and the general case gXM, where ‘X’ can take on any ‘x’ of the categorical AS):

$$CL23/F_{i}=\left\{ \begin{aligned} \theta_{Ref}\cdot e^{\eta_{i,CL23}} if AS=Ref, i.e. gNM \\ \theta_{Ref}\cdot\left( 1+\theta_{AS:0}\cdot X_{AS:0,i} \right)\cdot e^{\eta_{i,CL23}} if AS=0, i.e. gPM \\ \theta_{Ref}\cdot\left( 1+\theta_{AS:x}\cdot X_{AS:x,i} \right)\cdot e^{\eta_{i,CL23}} if AS=x, i.e. gXM \\ \theta_{Ref}\cdot\left( 1+\theta_{AS\geq2.5}\cdot X_{AS\geq2.5,i} \right)\cdot e^{\eta_{i,CL23}} if AS\geq2.5, i.e. gUM \end{aligned} \right.$$

In these models, $\theta_{Ref}$ represents CL23/F when AS = 2, i.e. the most frequent AS group translated as CYP2D6 normal metaboliser according to (14). θ_AS_ for continuous AS covariates and $\theta_{AS:x}$ for the five categorical AS covariates (excluding AS: 2, being the reference) describe the estimated effect of CYP2D6 activity (score) on CL23/F.

## 1.3 Advanced model evaluation diagnostics

**Covariate impact: Magnitude and precision** To draw inferences about clinical importance of parameter-covariate relationships, physiological plausibility, statistical (i.e. LRT) and clinical nonrelevance criteria (15,16) were evaluated concertedly. To infer about the clinical relevance of covariate effects, a forest plot was created to visually ascertain the magnitude and precision of estimated covariate effects (15,17) on model parameters CL20/F and CL23/F. 95%CIs as metric for parameter precision were derived applying sampling importance resampling (SIR) (18,19) . A covariate parameter estimate being within the area of ± 20% from the reference parameter value was considered as clinically irrelevant (clinical irrelevance criterion). In order to derive 95% CIs, the following SIR settings (proposal distribution and re-/sampling ratio) were selected and considered appropriate according to the resulting SIR diagnostic plots: As multivariate parametric proposal distribution, the variance-covariance matrix from the full covariate model was used and parameter vectors, M (samples) and m (resamples), were sampled and re-sampled applying a M/m ratio of 5000/1000.

**Goodness-of-fit and visual predictive check**  Goodness-of-fit plots and visual predictive checks (VPCs) were performed to assess the model performance of the developed joint tamoxifen-endoxifen PK model (Supplementary Figure 2). VPCs were faceted on relevant subgroups to assess the agreement of simulated median, intervals between 25^th^-75^th^ and 5^th^-95^th^ percentiles and observations over time after last dose for tamoxifen and endoxifen concentrations from the PK database. Visual predictive box-whisker plots of the simulated tamoxifen and endoxifen concentrations at steady-state were overlaid with the corresponding observed data points to compare the predicted and observed distributions across relevant subgroups.

# Supplementary Figures


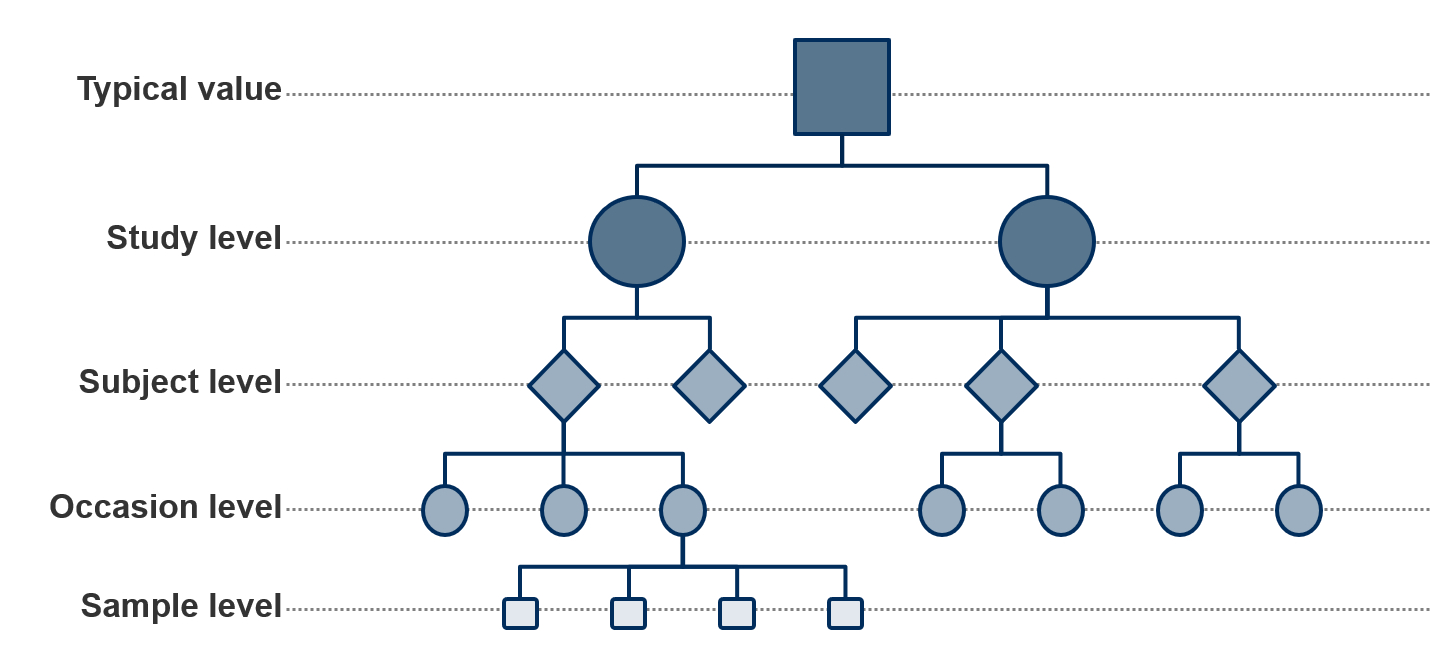


Supplementary Figure 1. Hierarchical structure for several levels of variability.


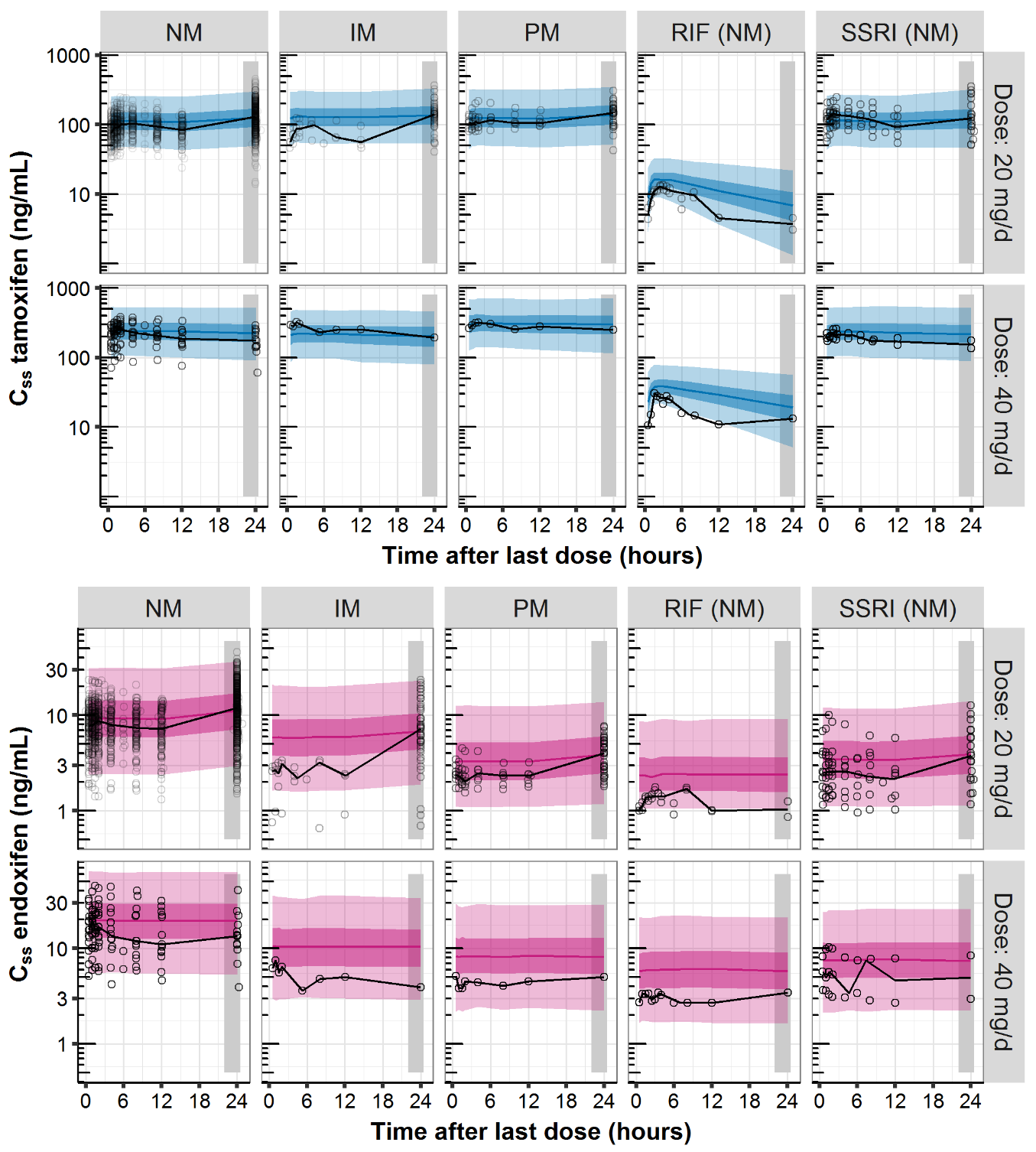


**Supplementary Figure 2.** Visual predictive check of (**top**) tamoxifen and (**bottom**) endoxifen concentrations over time after last dose at PK steady-state (*C_SS_*) stratified by dose and distinct sub-group (i.e. CYP2D6 metabolizer status, CYP perpetrator co-administration). Observed medians (black solid lines), s~~S~~imulated medians (colored solid lines), 25^th^-75^th^ (dark shaded area) and 5^th^-95^th^ (light shaded area) prediction intervals overlaid with the original tamoxifen observations from the PK database. *Vertical shaded area:* Predictive performance within this area (trough concentration phase around 24-hours) was most important. *NM/IM/PM:* CYP2D6 normal (incl. ultra-rapid)/intermediate/poor metabolizer; *RIF* *(NM)*: Rifampicin (strong CYP3A inducer) co-administration in NM; *SSRI (NM)*: Selective serotonine reuptake inhibitor (fluoxetine/paroxetine: strong CYP2D6 inhibitors) co-administration in NM.


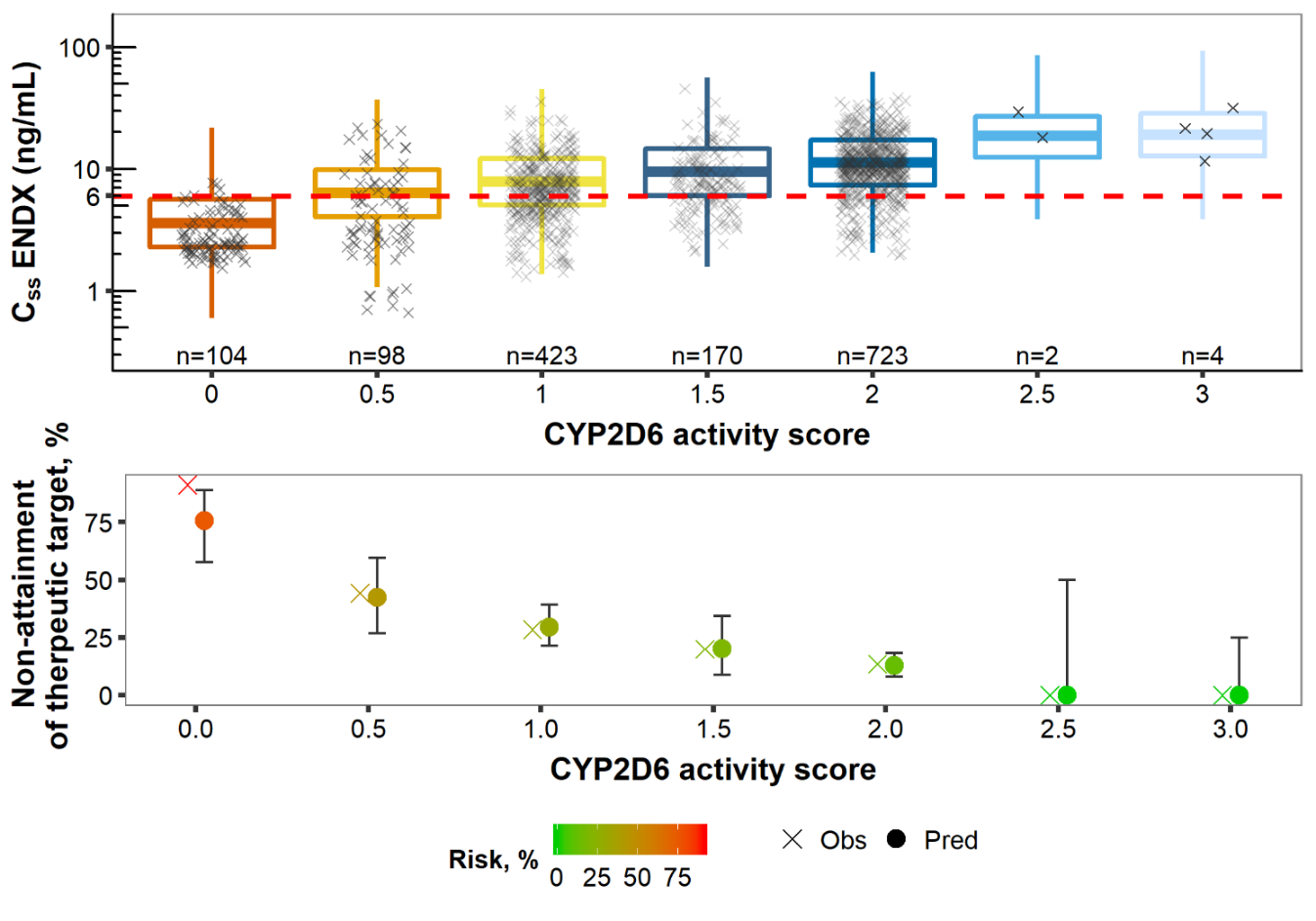


Supplementary Figure 3. Predicted (boxes and whiskers) and observed (X) dose-normalized concentrations at steady-state (C_SS_) of endoxifen, stratified by CYP2D6 activity score (top) and corresponding %non-attainment of therapeutic target endoxifen concentration (bottom). Box-whiskers: simulations (1000 replicates of observed dataset); black X: observed C_SS_ (Study 1-6); red horizontal dashed line: proposed endoxifen therapeutic threshold concentration (12); n: number of observed C_SS_ values; coloured ● and black whiskers: median and 95% confidence interval of predicted risk values; coloured X: observed risk values (Equation 5 in main text).

# References

1. Neven P, Jongen L, Lintermans A, Van Asten K, Blomme C, Lambrechts D, et al. Tamoxifen metabolism and efficacy in breast cancer: A prospective multicenter trial. Clin Cancer Res. 2018;24(10):2312–8.

2. Poppe A, Dieudonné A-S, Lintermans A, Laenen A, Blomme C, Lambrechts D, et al. Abstract P3-07-46: CYPTAM-BRUT 3: Endometrial thickness cannot be used as a marker for tamoxifen metabolization in postmenopausal breast cancer patients. Cancer Res [Internet]. 2016 Feb 15;76(4 Supplement):P3-07-46 LP-P3-07–46. Available from: http://cancerres.aacrjournals.org/content/76/4_Supplement/P3-07-46.abstract

3. de Graan A-JM, Teunissen SF, de Vos FYFL, Loos WJ, van Schaik RHN, de Jongh FE, et al. Dextromethorphan As a Phenotyping Test to Predict Endoxifen Exposure in Patients on Tamoxifen Treatment. J Clin Oncol. 2011 Jul;29(24):3240–6.

4. Teunissen SF, Rosing H, Koornstra RHT, Linn SC, Schellens JHM, Schinkel AH, et al. Development and validation of a quantitative assay for the analysis of tamoxifen with its four main metabolites and the flavonoids daidzein, genistein and glycitein in human serum using liquid chromatography coupled with tandem mass spectrometry. J Chromatogr B Anal Technol Biomed Life Sci. 2009;877(24):2519–29.

5. Binkhorst L, van Gelder T, Loos WJ, de Jongh FE, Hamberg P, Moghaddam-Helmantel IM, et al. Effects of CYP induction by rifampicin on tamoxifen exposure. Clin Pharmacol Ther. 2012;92(1):62–7.

6. Binkhorst L, Mathijssen RHJ, Ghobadi Moghaddam-Helmantel IM, de Bruijn P, van Gelder T, Wiemer EAC, et al. Quantification of tamoxifen and three of its phase-I metabolites in human plasma by liquid chromatography/triple-quadrupole mass spectrometry. J Pharm Biomed Anal [Internet]. 2011;56(5):1016–23. Available from: http://dx.doi.org/10.1016/j.jpba.2011.08.002

7. Binkhorst L, Bannink M, de Bruijn P, Ruit J, Droogendijk H, van Alphen RJ, et al. Augmentation of Endoxifen Exposure in Tamoxifen-Treated Women Following SSRI Switch. Clin Pharmacokinet. 2016;55(2):249–55.

8. Binkhorst L, Kloth JSL, de Wit AS, de Bruijn P, Lam MH, Chaves I, et al. Circadian variation in tamoxifen pharmacokinetics in mice and breast cancer patients. Breast Cancer Res Treat. 2015;152(1):119–28.

9. Dahmane EBA. Tamoxifen pharmacokinetics and pharmacogenetics in endocrine sensitive breast cancer patients. Thesis. Thèse de doctorat: Univ. Gnève, no. Sc. 4617 [Internet]. 2013. Available from: http://archive-ouverte.unige.ch/unige:33429

10. Ter Heine R, Binkhorst L, De Graan AJM, De Bruijn P, Beijnen JH, Mathijssen RHJ, et al. Population pharmacokinetic modelling to assess the impact of CYP2D6 and CYP3A metabolic phenotypes on the pharmacokinetics of tamoxifen and endoxifen. Br J Clin Pharmacol. 2014;78(3):572–86.

11. Dickschen K.; Thelen, K.; Lippert, J.; Hempel, G.; Eissing, T. K. W. Physiologically based pharmacokinetic modeling of tamoxifen and its metabolites in women of different CYP2D6 phenotypes provides new insight into the tamoxifen mass balance. Front Pharmacol. 2012;3.

12. Owen JS, Fiedler-Kelly J. Introduction to population pharmacokinetic/pharmacodynamic analysis with nonlinear mixed effects models. 1st ed. Hoboken, New Jersey: John Wiley & Sons, Ltd; 2014.

13. Bonate PL. Pharmacokinetic-pharmacodynamic modeling and simulation. 2nd ed. New York, USA: Springer Science + Business Media, LLC,; 2011.

14. Gaedigk A, Sangkuhl K, Whirl-Carrillo M, Klein T, Leeder JS. Prediction of CYP2D6 phenotype from genotype across world populations. Genet Med. 2017;19:69–76.

15. Ravva P, Gastonguay MR, Tensfeldt TG, Faessel HM. Population pharmacokinetic analysis of varenicline in adult smokers. Br J Clin Pharmacol. 2009;68(5):669–81.

16. Tunblad K, Lindbom L, McFadyen L, Jonsson EN, Marshall S, Karlsson MO. The use of clinical irrelevance criteria in covariate model building with application to dofetilide pharmacokinetic data. J Pharmacokinet Pharmacodyn. 2008;35(5):503–26.

17. Ito K, Murphy D. Tutorial: Application of ggplot2 to pharmacometric graphics. CPT Pharmacometrics Syst Pharmacol. 2013;2(10):1–16.

18. Dosne AG, Bergstrand M, Harling K, Karlsson MO. Improving the estimation of parameter uncertainty distributions in nonlinear mixed effects models using sampling importance resampling. J Pharmacokinet Pharmacodyn. 2016;43(6):583–96.

19. Dosne AG, Bergstrand M, Karlsson MO. An automated sampling importance resampling procedure for estimating parameter uncertainty. J Pharmacokinet Pharmacodyn. 2017;44(6):509–20.
